# Differential patterns of cross-reactive antibody response against SARS-CoV-2 spike protein detected for chronically ill and healthy COVID-19 naïve individuals

**DOI:** 10.1038/s41598-022-20849-6

**Published:** 2022-10-07

**Authors:** Mariliis Jaago, Annika Rähni, Nadežda Pupina, Arno Pihlak, Helle Sadam, Jürgen Tuvikene, Annela Avarlaid, Anu Planken, Margus Planken, Liina Haring, Eero Vasar, Miljana Baćević, France Lambert, Eija Kalso, Pirkko Pussinen, Pentti J. Tienari, Antti Vaheri, Dan Lindholm, Tõnis Timmusk, Amir M. Ghaemmaghami, Kaia Palm

**Affiliations:** 1 Protobios LLC, Tallinn, Estonia; 2 Department of Chemistry and Biotechnology, Tallinn University of Technology, Tallinn, Estonia; 3 North Estonia Medical Centre Foundation, Tallinn, Estonia; 4 Institute of Clinical Medicine, Psychiatry Clinic of Tartu University Hospital, University of Tartu, Tartu, Estonia; 5 Department of Physiology, Institute of Biomedicine and Translational Medicine, University of Tartu, Tartu, Estonia; 6 Center of Excellence for Genomics and Translational Medicine, University of Tartu, Tartu, Estonia; 7 Dental Biomaterial Research Unit (d-BRU), Faculty of Medicine, University of Liege, Liege, Belgium; 8 Department of Periodontology and Oral Surgery, Faculty of Medicine, University of Liege, Liege, Belgium; 9 Department of Anaesthesiology, Intensive Care and Pain Medicine, Helsinki University Hospital, Helsinki, Finland; 10 SleepWell Research Programme, Department of Pharmacology, University of Helsinki, Helsinki, Finland; 11 Oral and Maxillofacial Diseases, Helsinki University Hospital, University of Helsinki, Helsinki, Finland; 12 Translational Immunology Research Program, Department of Neurology, Neurocenter, Helsinki University Hospital, University of Helsinki, Helsinki, Finland; 13 Department of Virology, Medicum, University of Helsinki, Helsinki, Finland; 14 Department of Biochemistry and Developmental Biology, Faculty of Medicine, University of Helsinki, Helsinki, Finland; 15 Minerva Foundation Institute for Medical Research, Helsinki, Finland; 16 Immunology and Immuno-Bioengineering Group, School of Life Science, Faculty of Medicine and Health Sciences, University of Nottingham, Nottingham, United Kingdom; 17 Present Address: DXLabs LLC, Tallinn, Estonia

**Keywords:** Immunological memory, Translational research

## Abstract

Immunity to previously encountered viruses can alter response to unrelated pathogens. We reasoned that similar mechanism may also involve SARS-CoV-2 and thereby affect the specificity and the quality of the immune response against the virus. Here, we employed high-throughput next generation phage display method to explore the link between antibody immune response to previously encountered antigens and spike (S) glycoprotein. By profiling the antibody response in COVID-19 naïve individuals with a diverse clinical history (including cardiovascular, neurological, or oncological diseases), we identified 15 highly antigenic epitopes on spike protein that showed cross-reactivity with antigens of seasonal, persistent, latent or chronic infections from common human viruses. We observed varying degrees of cross-reactivity of different viral antigens with S in an epitope-specific manner. The data show that pre-existing SARS-CoV-2 S1 and S2 cross-reactive serum antibody is readily detectable in pre-pandemic cohort. In the severe COVID-19 cases, we found differential antibody response to the 15 defined antigenic and cross-reactive epitopes on spike. We also noted that despite the high mutation rates of Omicron (B.1.1.529) variants of SARS-CoV-2, some of the epitopes overlapped with the described mutations. Finally, we propose that the resolved epitopes on spike if targeted by re-called antibody response from SARS-CoV-2 infections or vaccinations can function in chronically ill COVID-19 naïve/unvaccinated individuals as immunogenic targets to boost antibodies augmenting the chronic conditions. Understanding the relationships between prior antigen exposure at the antibody epitope level and the immune response to subsequent infections with viruses from a different strain is paramount to guiding strategies to exit the COVID-19 pandemic.

## Introduction

The coronavirus disease 2019 (COVID-19) pandemic has unveiled the pathogenicity of SARS-CoV-2 with surges by the currently prevailing SARS-CoV-2 variant B.1.1.529 (described first in 2021)^1^ designated as Omicron displaying unusually large number of mutations and fast-spreading sublineages^2,3^. In general, clinical manifestations of SARS-CoV-2 infection range from asymptomatic and relatively milder, flu-like symptoms^4–6^ to long-lasting complications known as the post-COVID syndrome or long COVID^7,8^. This complex clinical picture along with confirmative studies that vaccination protects against severe forms of disease^9^ points to the immune system as a key factor in the control of SARS-CoV-2^10^.

The relative manifestation of symptoms in infected is likely attributable to the partial protection conferred by the pre-existing immune memory. The preference of the immune system to recall existing memory cells, rather than stimulate a de novo response when encountering a novel but closely related antigen is referred to as immune imprinting, historically known as original antigenic sin^11^. Overall, immune imprinting would lead to enhanced immunity, whereas established pre-immunity may also increase cross-reactive antibody response towards epitopes that are shared between the current and the previously encountered antigen^12,13^. Studies have observed cross-reactivity between endemic common cold human coronaviruses (HCoVs) and SARS-CoV-2^14–16^,whereas whether this cross-reactivity^17,18^ is beneficial or detrimental to COVID-19 disease is not clear^14,19–25^. Cross-reactivity through heterologous immunity may arise through recognition of identical antigenic epitopes shared by different pathogens, or through recognition of unrelated epitopes owing to cross-reactivity of individual T and B cell receptors^26^. Shifts in antibody response to respiratory syncytial virus, cytomegalovirus (CMV) and herpes simplex virus-1 (HSV-1) were noted in patients with severe COVID-19^27^. Cross-protective effects of non-COVID-19 vaccines against SARS-CoV-2 are currently tested in clinical trials for polio, measles-mumps-rubella, influenza, and Bacillus Calmette–Guérin vaccines (rev in^28^) with promising pre-publication findings^29^. Collectively these data demonstrate that people at the stage of the current pandemic carry heterogeneous, immune-imprinted repertoires derived from their distinctive histories of infection and vaccination.

Given that the infections with SARS-CoV-2, in particular with its Omicron variants have become so common, it is likely that these confer boosting to the prior immune repertoire. Reports are emerging on findings of IgG autoantibodies in COVID-19 patients with a significant subset of patients developing new-onset autoantibodies^30–33^ that could place them at risk for progression to autoimmunity. Studies reporting on myocarditis after receiving mRNA vaccines against COVID-19^34–38^ suggest molecular mimicry between the vaccine product and self-antigens as an underlying mechanism^39^.

Given that the relationships between prior antigen exposure, through infection or vaccination with SARS-CoV-2, and the immune responses to subsequent infections with emerging viruses is still incompletely understood, but is of paramount importance to exit the COVID-19 pandemic, we employed an unbiased approach of next generation peptide phage display mimotope variation analysis (MVA)^40,41^, to delineate cross-reactive immunity hallmarks on SARS-CoV-2 S glycoprotein in COVID-19 naïve subjects. Using samples from both COVID-19 naïve individuals and patients with a COVID-19 diagnosis, we identified pre-existing antibody response to multiple S protein sites by using recombinant S protein subunits of SARS-CoV-2 that was increased in patients with severe COVID-19 disease. Among these, three epitopes with cross-reactivity to SARS-CoV-2 S were features of underlying acute and/or chronic clinical conditions. Thus, highlighting the risk for chronic condition exacerbation following SARS-CoV-2 S protein exposure due to infection or vaccination.

## Star★methods

### Key resources table

**Table:** 

Reagent or resource	Source	Identifier
**Antibodies**		
Human IgG reference pool	Sigma-Aldrich	Cat#i4506
Rabbit anti-human IgG (H&L) (HRP)	Abcam	Cat#ab6759
**Biological samples (serum, plasma)**		
COVID-19	Tartu University Hospital	COVID-19
Coronary artery disease	Helsinki University Hospital	CVD (CAD)
Myocardial infarction	North Estonia Regional Hospital	CVD (MI)
T2D and T2D with foot ulcers (DFU)	University of Liege	T2D
Multiple sclerosis	Helsinki University Hospital	MS
Breast cancer	Helsinki University Hospital	BC
First episode psychosis, schizophrenia	Psychiatry Clinic of Tartu University Hospital, Helsinki University Hospital	ND (FEP; SZ)
Healthy donor	North Estonia Medical Blood Centre	HC
**Chemicals, peptides, and recombinant proteins**		
SARS-CoV-2 spike protein S1 subunit	Icosagen	Cat#P-305-100
SARS-CoV-2 spike protein S2 subunit	Icosagen	Cat#P-306-100
Ph.D.™-12 phage display peptide library (modified from original library)	New England Biolabs	Cat#E8110S
**Critical commercial assays**		
Catalysed signal amplification (CSA) system II, biotin-free, HRP, DAB+	Dako (Agilent)	Cat#K1497
Anti-CMV ELISA (IgG)	EUROIMMUN	Cat# EI 2570-9601 G
Anti-EBV-CA ELISA (IgG)	EUROIMMUN	Cat# EI 2791-9601 G
**Software and algorithms**		
SPEXS2 algorithm	Courtesy of Egon Elbre	https://github.com/egonelbre/spexs2
ImageJ v. 1.53a	Schneider et al., 2012	https://imagej.nih.gov/ij/
RStudio v. 1.3.959	RStudio Team, 2020	https://www.rstudio.com
R “tidyverse” packages	Wickham et al., 2019	https://doi.org/10.21105/joss.01686
R “HPAanalyze” package	Tran et al., 2019	https://doi.org/10.1186/s12859-019-3059-z
R “ggpubr” package	Courtesy of Kassambara	https://CRAN.R-project.org/package=ggpubr
R “ggbeeswarm” package	Courtesy of Clarke and Sherrill-Mix	https://CRAN.R-project.org/package=ggbeeswarm
MS Office Excel 2016	Microsoft Corporation	https://www.microsoft.com
Adobe Photoshop CS4 version 11.0	Adobe Systems Inc	https://www.adobe.com
**Other**		
Protein G magnetic beads	New England Biolabs	Cat#S1430S
Immune epitope database	Immune Epitope Database	https://www.iedb.org
Human protein atlas v.20.0	Uhlen et al., 2015^42^	https://www.proteinatlas.org
UniProtKB human reference proteome	^43^	https://www.uniprot.org/proteomes/UP000005640; ID: UP000005640
SARS-CoV-2 proteome sequences	^44^	https://www.viralzone.expasy.org/8996

## Experimental model and subject details

### Ethics declarations

The study was conducted in accordance with the guiding principles of the Declaration of 182 Helsinki and the study participants gave written informed consent before enrolment. The sample data on myocardial infarction (MI), breast cancer (BC) and schizophrenia (SZ) samples are described in detail in Pupina et al. 2022^45^. The use of multiple sclerosis (MS) and coronary artery disease (CAD) cohort samples was approved by the regional ethics committees (Dno 83/13/03/01/2013^40^ and licence no 106/2007^46^, respectively). Use of samples from type II diabetes (T2D) patients was approved by the Ethics Committee of University Hospital of Liege (permit no 2018/78). First-episode psychosis (FEP) cohort and samples from COVID-19 patients were used with the approval of the Ethics Committee of the University of Tartu (licenses no. 362/T-3 and 312T-2, respectively) and have previously been studied in^47,48^. Healthy blood donor (HC) samples were procured from the North Estonia Medical Blood Centre (Tallinn, Estonia) and approved with study licence TAIEK no 1045.

### Clinical cohorts

The antibody immune profiles were generated by MVA from SARS-CoV-2 naïve individuals (n = 538, Table 1), comprising patients with different clinical diagnoses (n = 276) and healthy controls (n = 262). COVID-19 naïve blood samples were collected prior to SARS-CoV-2 emergence (between April 2010 to Dec 2018) and chosen to represent a cross-section of the general uninfected, unexposed, unvaccinated population. Cardiovascular disease (CAD^46^ and MI^45^) and neuropsychiatric disease (FEP and SZ^45,47,48^) samples are matched with control samples (Table 1) by the design of the original studies. Type II diabetes (T2D), multiple sclerosis (MS) and breast cancer (BC) sub-cohort patient samples have been analysed against selected controls samples (Table 1). In addition, time-series samples from six patients with COVID-19 diagnoses were analysed by MVA, and samples from two of these patients were analysed by dot-ELISA methods (Table S1). These samples were initially collected in the hospital emergency room (ER) and consecutive time-series samples were collected in the stationary care unit during disease progression or recovery process.

**Table 1:** Descriptions of clinical cohorts of COVID-19 naïve individuals.

No	Cohort (abbreviation)	Study group	Group size (n)	Gender	Age (mean ± standard deviation (SD)), years	Origin	Case or ctrl	Previous studies
**Cardiovascular diseases (CVD, with comorbidities including obesity, hypertension, diabetes)**								
1	CAD	No-CAD	32	17M/15 F	60.2 ± 8.3	Finland	Ctrl	^46^
		Stable-CAD	32	26M/6F	64.3 ± 8.4	Finland	Case	^46^
		Acute coronary syndrome	32	24M/8F	61.3 ± 8.2	Finland	Case	^46^
2	MI	HC	61	25M/36F	46.2 ± 15.5	Estonia	Ctrl	^45^
		MI	50	29M/13F/10 not available (NA)	66.8 ± 12.6	Estonia	Case	^45^
3	T2D and foot ulcer	T2D	25	21M/4F	69.0 ± 8.7	Belgium	Case	None
**Autoimmune disease**								
4	MS	MS	20	4M/16F	32.3 ± 7.8	Finland	Case	^40,45^
**Cancer**								
5	BC	BC	57	0M/57F	55.7 ± 7.2	Finland	Case	^45^
**Neuropsychiatric disorders (ND)**								
6	FEP	HC	30	15M/15F	24.0 ± 6.1	Estonia	Ctrl	^47,48^
		FEP	30	16M/14F	25.6 ± 4.9	Estonia	Case	^40,47,48^
7	SZ	HC	30	12M/18F	42.1 ± 18.2	Finland	Ctrl	^45^
		SZ	30	12M/18F	41.9 ± 18.4	Finland	Case	^45^
**Healthy blood donors**								
8	Donors	HC	109	64M/45F	40.6 ± 11.6	Estonia	Ctrl	^40,45^

## Method details

### Mimotope-variation analysis

For qualitative and quantitative characterisation of humoral immune response, we used an in-house developed mimotope-variation analysis (MVA) method as described previously^40,41^. In brief, modified random 12-mer peptide phage library (Ph.D.-12, New England Biolabs) was used. Two µl of serum/plasma samples was precleared to plastic and *E. coli*/wild type M13 phage particles. After preclearing, serum/plasma samples were incubated with 2.5 μl library (~ 5 × 10^11^ phage particles) and phage-immunoglobulin G (IgG) antibody complexes were recovered using protein G-coated magnetic beads (S1430S, New England Biolabs). Captured phage DNA was analysed by next generation sequencing (Illumina HiSeq, 50-bp single end reads) with barcoded primers for sample multiplexing. To evaluate the reproducibility of the data, we compared peptide abundance in two replicates using Pearson correlation coefficient, which was 0.985 (p < 0.0001) (Fig. S1). Obtained sequences were bioinformatically cleaned of sequencing errors and known artefacts, yielding peptide profiles for further analysis.

### Clustering of immunodominant peptide antigens

Peptides in a sample dataset cleaned of sequencing errors and known artefacts were normalised to 3 million reads (RPM units). SPEXS2 exhaustive pattern search algorithm^41^ was used to cluster similar peptides and reveal recognition patters (epitope consensuses) that were enriched in studied peptide sets. The identification of core consensus sequences was either performed discriminatorily (see below for details), where selected peptide sets were studied for enrichment compared to control group, or non-discriminatorily (see below for details), where enrichment was identified compared to a random-generated peptide set (hypothesis-free). The decision of whether to compare sample peptides to random-generated reference peptide sets or to peptides from matched controls samples was determined by the clinical question of the study^40,45,46^with parameters chosen to maximise the number of epitope consensuses in the output. In brief, for each clinical group, 100,000s of distinct peptide antigens were used as input, from which 1000s of distinct core epitopes were identified with SPEXS2 algorithm. The output of distinct core epitopes generated for each of the sub-cohorts (CAD, MI, T2D, BC, FEP, SZ, MS) were combined together and further analysis was carried out across the whole study cohort. Altogether a total set of core epitopes (n = 22,949) was generated, for the entire cohort including data from samples of different age groups, genders and clinical background. All the 22,949 consensus epitope sequences were aligned to primary sequence of the SARS-CoV-2 spike glycoprotein (S) (UniProtKB code: P0DTC2).

### Non-discriminatory clustering

The generation of distinct consensus epitopes (n = 8088) for CAD cohort is described in detail in^46^. The generation of distinct consensus epitopes (n = 4019) for MI cohort is in^45^.

For analysis of T2D with foot ulcer condition, most abundant and shared peptides from MVA immunoprofiles were selected with criteria: peptide must be present in ≥ 10 repeats in one sample; and must be present in ≥ 2 samples. The resulting peptide set was compared with random-generated peptide set of same length and enriched consensuses were identified using SPEXS2 algorithm, using hypergeometric p-value < 10^–7^ and motif to be present in ≥ 4 distinct peptides. As a result, epitopes (n = 1169) with ≥ 4 fixed amino acid positions were identified.

For cancer-related immunoprofiles, patients with surgically removed BC (denoted as BC), top 900 most abundant peptides from each sample were screened separately for epitope consensus identification (≥ 5 fixed amino acid positions). These identified from ≥ 2 samples were extracted for further analysis and motifs more detected in BC were selected. Additionally, peptides from a large non-unique dataset (shared in ≥ 2 samples) that did not contain any consensuses identified by the above-mentioned approach, were examined separately. Peptides present in ≥ 4 samples (> 10%) in BC group were extracted and common consensus sequences were identified with SPEXS2 tool (with hypergeometric p-value < 10^–3^, ≥ 5 fixed amino acid positions, epitope consensus to be present in ≥ 4 unique peptides). Altogether, distinct consensus epitopes (n = 1,014) were selected for further analysis.

### Discriminatory clustering

Within FEP and SZ cohorts, most abundant and shared group-specific peptide antigens were extracted for each of the control and case groups, based on criteria: the chosen peptide must be present in ≥ 10 repeats in one sample; and must be present in ≥ 10% of the samples within the given group. Core consensus epitopes were defined for each group independently with SPEXS2 as motifs that were more enriched when compared to at least one of the 3 different reference sets: (1) random-generated reference set of same length, where amino acids were scrambled within-peptides; (2) random-generated reference set of same length, where amino acids were scrambled within amino acid positions- and across peptides; and (3) the top abundant peptide set from the control group. Distinct consensus epitopes identified from SPEXS2 analyses were selected, based on criteria: hypergeometric p-value < 10^–6^ (FEP), < 10^–5^ (HC of FEP), < 10^–8^ (SZ), or < 10^–6^ (HC of SZ); consensus epitope present in ≥ 4 distinct peptides; ≥ 4 fixed amino acid positions. Epitope consensuses matching these criteria were selected for FEP (n = 228), SZ (n = 1785), HC of FEP (n = 1935), HC of SZ (n = 760).

Within MS group, subgroups with or without initial optic neuritis diagnosis were analysed separately. The topmost abundant peptides from both subgroups were extracted with criteria: peptide count ≥ 5 in ≥ 1 sample. Using SPEXS2 the peptide sets were compared to top peptide set of age- and sex-matched controls with no MS diagnosis^40^ using criteria: hypergeometric p-value < 10^–7^; epitope consensus to be present in ≥ 4 distinct peptides and have ≥ 4 fixed amino acid positions. As a result, distinct epitopes (n = 3,500) were identified for MS group.

### Epitope prediction of SARS-CoV-2 S

The primary sequence of SARS-CoV-2 S (P0DTC2) was obtained from www.viralzone.expasy.org/8996 (date accessed 25.03.2020). To predict immunogenic regions on S, we followed two complimentary alignment approaches. First, primary sequence of SARS-CoV-2 S protein was scanned with distinct epitope consensus (n = 22,949) with the criterion of  ≥ 4 exact-matching amino acid positions. Random reference profile was generated by scanning with shuffled-sequence motifs (≥ 4 exact positions) in order to qualitatively assess alignment enrichment on local protein regions. An additional random alignment distribution was simulated with 3 independent alignments with scrambled motifs in order to assess statistical significance of specific alignment results. Specific alignments with values above 97.5th percentile (two-tailed) of simulated distribution were considered statistically significant (*p < 0.025, **p < 0.005, ***p < 0.0005). Two regions (amino acid positions 623–633 and 708–713) were excluded based on lower count of unique peptide epitopes (< 200) or insufficient specific/random ratio (< 3 positions with ratio ≥ 2) values. Secondly, to identify regions with less prevalent yet sufficiently high alignment enrichment results, an additional approach was taken. For the epitope consensuses, the number of fixed amino acids for alignments was 4. Therefore, on a protein region of 20 amino acids, the probability of exact match with this region was 17/20^4^ = 1.1 × 10^–4^. With an input of all epitope consensuses (n = 22,949), the number of theoretical exact alignments to any 20-amino acid region would be 2.4. Therefore, to identify potential immunogenic regions, the threshold for significance was set to ≥ 3 of exact-aligned epitope consensuses. Based on this criterion, epitopes S1.4 and S2.1 were included with the other described immunogenic regions. The composition of top 10 most abundant peptides representing the 15 defined S epitopes across the cohort of 538 samples is shown in Table S2.

Alignment profiles on the primary sequence of SARS-CoV-2 S protein were generated using custom Excel VBA scripts and MS Office Excel, and visualised using R “tidyverse” packages^49^. The data was visualised using centred weighted moving averages across 9 amino acid positions. The displayed value (*value*) was calculated per each amino acid position (*n*), taking into account the raw values (*a*) of given and four adjacent positions in both directions and multiplying those with weights (from 1 to 5):

$$value= \frac{1*\left({a}_{n-4}+{a}_{n+4}\right)+2*\left({a}_{n-3}+{a}_{n+3}\right)+3*\left({a}_{n-2}+{a}_{n+2}\right)+4*\left({a}_{n-1}+{a}_{n+1}\right)+5*{a}_{n}}{25}$$

### Sequence alignment

#### Individual sample peptide alignment

For the analysis of individual peptide alignments of samples that were used in dot-ELISA (n = 9), all peptide sequences underlying the 15 MVA-predicted epitopes were aligned to SARS-CoV-2 S protein primary sequence, taking into account the different abundance values of peptides in individual immunoprofiles. Alignment was performed with ≥ 4 matching amino acid positions, ratio of specific/random alignment (with shuffled peptide sequences) was calculated for each amino acid position on SARS-CoV-2 S protein primary sequence, and data was visualised as heatmaps using centred weighted moving averages of ratios across nine amino acid positions.

#### Human pathogen and human proteome alignment

Reference proteins of common human viruses were obtained from UniProtKB with keywords “taxonomy: “Viruses [10239]”” + “host: human” + “reviewed: yes” (date accessed: 10.11.2020, altogether 6114 viral proteins). Human reference proteome was obtained from UniProt Proteome database (Proteome ID: UP000005640, date accessed: 16.11.2020, altogether 75,069 sequences).

Constructed viral protein and human protein databases were first scanned with 15 SARS-CoV-2 S protein epitope representative motifs. Using custom Excel VBA scripts a reference set of viral and human proteins that contained at least 1 predicted S protein epitope was generated. 7.5% (458) of human-associated virus proteins and 6.3% (4,968) of human proteome sequences matched ≥ 1 MVA predicted S protein epitope(s). Next, the primary sequences of reference set proteins from Swiss-Prot (excluding TrEMBL) were aligned with MVA captured peptides containing these 15 S epitopes (900 most abundant peptides per each defined epitope across 538 samples) by using standalone BLAST + *blastp* function (v. 2.11.0) with the following criteria: (i) word size: 2, (ii) gap open penalty: 9, (iii) gap extend penalty: 1, (iv) substitution matrix: PAM30, (v) threshold: 11, and by disallowing composition-based statistics correction. E-value threshold for human virus proteins was set to 200,000, while E-value threshold for human proteome alignment was set to 2000. Output was further curated to select for the highest scoring target alignments of the 15 epitopes of SARS-CoV-2 S based on E-value (< 0.05, except for human coronavirus proteins, where E-value > 0.05 was allowed) and identity (> 48%, i.e. at least 5 matching amino acid positions in the 12-mer peptide for viral proteins and > 52%, i.e. at least 5 matching amino acid positions from the 12-mer peptide for human proteins) (Tables S3 and S4).

The alignment data were visualised using R statistical programming (v. 4.0.2, https://www.R-project.org/) and RStudio environment (http://www.rstudio.com/). Violin plots were produced and visualised using R “tidyverse” packages^49^.

Human Protein Atlas (HPA, v. 20.0, http://www.proteinatlas.org)^42^ gene expression data was used to visualise the expression of human proteins that aligned with SARS-CoV-2 S protein epitope peptides. “HPAanalyze” R package^50^ was used to access gene expression data of normal tissue from HPA repository. Expression data deemed “Uncertain” by the “Reliability” parameter were excluded and data was visualised with “ggplot2”^51^.

### Modelling immune response patterns in COVID-19 naïve subjects

IgG-bound peptide abundance values of 15 epitopes were calculated for each sample in the COVID-19 naïve cohort (n = 538) and normalised per epitope with the 97.5% percentile value (to allow for comparison of epitopes). Subjects with a relatively higher response (normalised value > 0.5) to at least two epitopes on S protein (of the 15) were grouped into the “high” group, whereas others to the “low” group.

### Visualisation of data from published data sets

B cell epitopes for the SARS-CoV-2 S protein for naïve individuals were derived from Grifoni et al. study^52^, for COVID-19 from several published studies^27,53–57^. Amino acids of SARS-CoV-2 S protein important for binding of human ACE2 have been reported by different groups^58^ and anti-spike RBD neutralising antibody CR3022 discontinuous epitope were reported by ter Meulen^59^ and Rogers and colleagues^53^. Domains of SARS-CoV-2 S protein were accessed from^54,60^. Raw data of PEPperCHIP^®^ SARS-CoV-2 Proteome Microarray is from Schwarz et al., 2021^61^. Reported epitopes in Immune Epitope Database (IEDB) were accessed on 11.07.2022 with the following criteria: *Organism*—SARS-CoV2 (ID:2697049); *Antigen*—Spike glycoprotein (PODTC2); *B cell Assay*—Outcome "Positive" and Neutralization" (Table S5).

### Dot-ELISA (CMV and EBV epitopes)

Independent validation experiments for IgG immunoreactivity measured by MVA were performed with dot-ELISA experiments. Peptides containing CMV- and EBV-specific epitopes previously described in^40^ were printed as follows. Recombinant phages displaying in the N-terminus of the pIII of M13 peptides of interest, specifically (TLPMDTSPRAHW containing epitope of viral capsid antigen (VCA) p18 of Epstein-Barr virus (EBV)) (*specific*) and TLPMDASPRAHW (control). In addition, peptides containing epitope of glycoprotein B (gB) of human cytomegalovirus (CMV) NETIYNTTLKYGGGGDYKDDD(LYS(BIOTIN)) were synthesised by Genescript (US). For dot-ELISA, peptides or peptide-displaying phages printed onto nitrocellulose filter pads (Amersham Bioscience) as duplicates by SpotBot^®^ 4 (Arrayit) were exposed to human precleared sera/plasma (dilution 1:100 for MS sub-group samples and 1:50 for CAD sub-group samples) for 1 h at room temperature and then incubated with rabbit anti-human IgG (HRP) (Abcam, ab6759; dilution 1:1000) as a secondary antibody. Images were scanned using EttanTM DIGEImager (GE Healthcare Life Sciences). All printed dot intensities were calculated and analysed further as averages of duplicates. With CAD samples, n = 28 samples were assayed for seroreactivity to epitope on gB of CMV and n = 54 samples for VCA p18 of EBV. Seroreactivity to epitope on gB of CMV was calculated by subtracting the background intensity of the dot and displayed in arbitrary units (AU). Seroreactivity to epitope on VCA p18 of EBV was calculated as the difference between signals of specific vs control phage (signal difference, in AU) in the MS cohort, whereas in the CAD cohort as the ratio between signals of specific vs control displayed epitopes (signal ratio, in AU).

### Dot-ELISA (spike subunits)

Spike protein fragments of S1 subunit (amino acids 14–681, cat no. P-305–100), S2 subunit (aa 693–1218, cat no. P-306–100) and RBD (aa 319–541, cat no. P-307–100) (Icosagen AS) were diluted in 1× PBS (pH 7.4), blotted onto nitrocellulose membranes (50 ng of protein per dot) (Amersham Biosciences, cat no. RPN 1520D) and blocked with 5% non-fat dried milk powder (DMP) (Applichem, cat. no. A0830) in 1xPBS-0.05%-Tween20 for 1 h at room temperature. For linear epitope analysis, spike protein subunits S1, S2 and RBD were denatured in 4 M Urea (Applichem, cat. no. A8113) for 1 h at room temperature prior to blotting. Sera/plasma samples (1:50 dilutions) were pre-treated overnight with 1:2 solution of *E. coli* phage lysate in 2.5% DMP in 1xPBS-0.05%-Tween20 at + 4 °C to reduce non-specific signals. Blocked nitrocellulose membranes (5% DMP in 1xPBS-0.05%-Tween20, 1 h at RT) were washed with 1xPBS-0.05%-Tween20 and incubated for 4 h at room temperature with human sera/plasma sample dilutions to form SARS-CoV-2 S protein-specific immunoglobulin and S subunit complexes. After washes, membranes were incubated with 1:1000 rabbit anti-human IgG HRP-conjugated secondary antibody (Abcam, cat no. ab6759) in 2.5% DMP in 1× PBS-0.05%-Tween20 for 1 h at room temperature. Signal amplification system (Dako, CSA II System kit, cat no. K1497) was used for sensitivity enhancement with reagents diluted to either 1:10 for amplification reagents or to 1:100 for DAB chromogen in 1× PBS-0.05%-Tween20 buffer. Membranes were digitally scanned and signals quantified using ImageJ software (version 1.53a)^62^. The averages from two technical replicate dots were calculated per each experiment (n = 3) and intensity values were represented as proportional to a COVID-19 positive case (P1#1, Table S1). Averages ± SEM of three independent experiments are represented on figures.

### ELISA

Anti-CMV and anti-EBV serostatus was measured from serum/plasma samples with ISO/IEC-17025:2017 accredited methods. In brief, serological analyses were performed with anti-CMV IgG ELISA method (EUROIMMUN EI 2570-9601 G) and with anti-EBV CA IgG ELISA method (EUROIMMUN EI 2791-9601 G) according to manufacturer’s specifications. Absorbance was measured at 450 nm with SpectraMax Paradigm (Molecular Devices). Altogether serum or plasma samples of 199 subjects from the clinical cohorts of healthy donors (n = 83), MS (n = 20), or CAD (n = 96) were analysed for anti-CMV IgG antibodies and 241 subjects from the clinical cohorts of healthy donors (n = 19), MS (n = 20), FEP (n = 60), SZ (n = 46), or CAD (n = 96) were analysed for anti-EBV CA IgG antibodies.

## Quantification and statistical analysis

### Statistical analysis

All statistical analyses (Pearson correlation calculation, Mann–Whitney U test, multiple linear regression analysis) were done using R statistical programming and RStudio environment (https://www.R-project.org/; URL: http://www.rstudio.com/). Boxplot graphs were produced and visualised using R “tidyverse” packages^49^, “ggbeeswarm”, and “ggpubr”^63,64^. On boxplots, the upper, middle and lower boxplot lines represent the 75th, 50th and 25th percentiles, while whiskers represent the largest or smallest value within 1.5 times interquartile range above the 75th percentile or below the 25th percentile, respectively and *outer dots* indicate outliers. Reported p-values were not adjusted for multiple comparisons, as this study was viewed as a hypothesis-free approach with an emphasis on discovering new relationships in a retrospective cohort.

## Results

### Cross-reactive immune response to SARS-CoV-2 spike protein in COVID-19 naïve people

We used a high throughput random peptide phage display method (MVA)^33,34^ to investigate potential cross-reactive antibody epitopes on SARS-CoV-2 S antigen in a cohort of SARS-CoV-2 unexposed (= COVID-19 naïve, also unvaccinated) individuals (n = 538, Table 1). Our discovery cohort of COVID-19 naïve included sera samples collected before 2017 from both, healthy individuals (Ctrl) and people diagnosed with various acute illnesses and chronic conditions (Case) to reflect the overall diversity of general population (Table 1). The mean age in case sub-cohorts of adults varied from 24 to 69 years, and the proportion of men varied between 20 and 84% for most sub-cohorts (Table 1).

**Figure 1:**
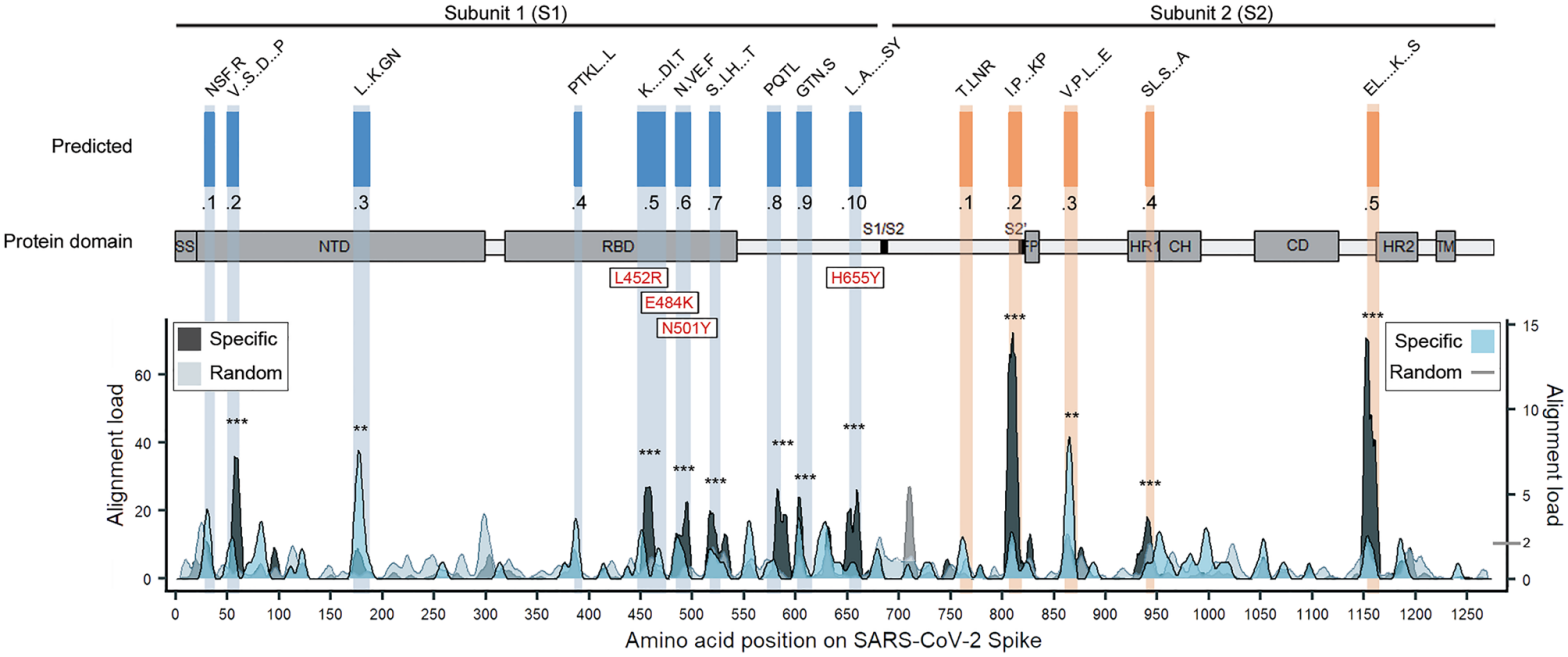
MVA-defined epitopes on SARS-CoV-2 spike protein. Alignment profiles of the most abundant and common immune response features from MVA immunoprofiling data of COVID-19 naïve subjects (n = 538), including altogether 22,949 unique epitopes characterising core motif sequences (hypergeometric p-value < 10^–3^ for core epitope recognition) on the primary sequence of SARS-CoV-2 S protein (UniProtKB code: P0DTC2). The abundance of aligned core epitope sequences (black Specific, primary y-axis) in peaks was significantly higher (**p < 0.01, ***p < 0.001) compared to random alignment (light gray random, primary y-axis). Of aligned core epitopes, 111 were with exact matches in all amino acid positions (blue specific, secondary y-axis). Regions of primary sequence with alignment load of > 2 motifs (the calculated theoretical random) (gray random line, secondary y-axis) were considered as potentially immunogenic and included in further analysis. Alignment profiles were visualised as centred moving averages across 9 amino acids. 15 epitopes (designated S1.1….10 on subunit 1 of S (S1) and S2.1….5 on subunit 2 of S (S2)) from MVA data analysis (predicted) were defined on SARS-CoV-2 S protein (x-axis) with representative consensus sequences shown. Common mutations observed in emerging SARS-CoV-2 variants were mapped to MVA-identified S epitopes and highlighted with red labels. Most common variants with enhanced transmissibility of SARS-CoV-2 variants with L452R, E484K/Q, and H655Y mutations^99,109,123^ encompass epitopes in S1.5, S1.6, or S1.10 respectively. Primary y-axis—abundance of specific- vs random-aligned sequences with ≥ 4 matching positions (includes also exactly-matching sequences). Secondary y-axis—abundance of aligned motif sequences with all positions matching. SARS-CoV-2 S protein domains are adapted from Wrapp et al.^54^ with additional information on RBD from Yuan et al.^53^. *SS* signal peptide ^1–12^; *NTD* N-terminal domain (13–303); *RBD* receptor binding domain (319–542); *S1/S2* S1 subunit end and S2 start site (683–686); *S2’* S2’ protease cleavage site (815–816); *FP* fusion peptide (816–833); *HR1* heptad repeat 1 (908–985); *CH* central helix (986–1035); *CD* connector domain (1076–1141); *HR2* heptad repeat 2 (1163–1202); *TM* transmembrane domain (1214–1234).

Using MVA we defined 15 highly antigenic epitopes on the S protein, of which ten were on subunit 1 (S1) and five on subunit 2 (S2) (Fig. 1 and Table 2). The majority of these epitopes (epitopes S1.1 to S2.4) were exposed on the exterior surface of SARS-CoV-2 S trimer (Fig. S2A). Seven of the 15 identified epitopes were partially overlapping with epitopes previously reported for COVID-19 unexposed individuals^52^ with an average overlap of 60% per epitope (Table 2). Epitopes S2.2 and S2.5 colocalised precisely with antigenic determinants reported by others, whereas epitope S1.8 extended to a more C-terminal region (amino acids 570–582) (Table 2). Furthermore, almost half of resolved epitopes (S1.8, S1.9, S1.10, S2.1, S2.2, S2.3, and S2.5) mapped to antigenic regions of S against which immune response has been detected in asymptomatic, mild, and severe COVID-19 cases (Table 2 and^27,55–57^). In good agreement with published data from SARS-CoV-2 proteome-based peptide arrays^61^, we found that linear peptides from these studies that contained our resolved epitopes on S protein showed differential seroreactivity in naïve, mild and severe COVID-19 samples (Fig. S3).

**Table 2:** The epitopes identified by MVA on SARS-CoV-2 spike glycoprotein are validated by using external data showing partial overlap with antigenic domains reported for COVID-19 naïve and diseased and with neutralising epitopes.

Epitope identification (a)	Amino acid position (b)	Sequence (c)	Representative epitope (d)	Data on antigenic regions from other studies (e)				S mutants of variants of SARS-CoV-2 (f)
				Bio-informatic predictions in COVID-19 naïve	Serostudies		IEBD: B cell neutralising antibody response	
					COVID-19 naïve	COVID-19 diseased		
**S1** **subunit**								
S1.1	26–34	PAYTNSFTR	NSF.R				P26	P26S^124^
S1.2	47–58	VLHSTQDLFLPF	V..S..D…P				–	Q52R^124^
S1.3	170–185	YVSQPFLMDLEGKQGN	L..K.GN				178–185	
S1.4	384–390	PTKLNDL	PTKL..L				384–390	
S1.5	445–471	VGGNYNYLYRLFRKSNL-KPFERDISTE	K….DI.T	469–483^52^			445–471	G446S^**O:A1,A3**^^2,71^ L452M^**OA2**^^71^ L452Q^**O:A2**^ ^71^ L452R ^**O:A4,5**^^71^ L452R/Q*^127^ G446S^**O**^^2^
S1.6	481–495	NGVEGFNCYFPLQSY	N.VE.F	469–483^52^			481–495	E484K/Q*^123,127^ E484A^**O**^^2^ F486V^**O:A5**^^71^ F490S^124^ Q493/K^**O**^^2^
S1.7	514–523	SFELLHAPAT	S…LH…T				514–523	
S1.8	570–582	ADTTDAVRDPQTL	PQTL		550–570^27^	553–570^55^ N** 550–570^27^ 532–588^27^ 560–616^27^	A570	A570D^124^
S1.9	599–612	TPGT**N**TSNQVAVLY	GTN.S	592–620^52^		588–644^27^ 560–616^27^	–	
S1.10	650–660	LIGAEHV**N**NSY	L..A…..SY	652–661^52^		655–672^56^ N** 616–672^27^	H655	H655Y^128^*^O^^124^ N658S ^**O:A5**^^71^
**S2 subunit**								
S2.1	757–768	GSFCTQLNRALT	T.LNR	757–769^52^		766–785^27^	–	N764K^**O**^^125^
S2.2	804–815	QILPDPSKPSKR	I.P…KP		810–816^57^ 785–805^27^ 810–830^27^	809–826^55^ N** 787–822^56^ 810–816^57^ 785–805^27^ 810–830^27^ 812–868^27^	–	
S2.3	858–869	LTVLPPLLTDEM	V.P.L…E	867–880^52^		812–868^27^	–	T859N^126^
S2.4	937–944	SLSSTASA	SL.S…A	915–946^52^			–	
S2.5	1151–1161	ELDKYFK**N**HTS	EL….K…S	1157–1164^52^	1146–1166^27^	1147–1158^56^ 1146–1166^27^	1148–1158	

The following information is given in columns: unique identification (a), amino acid position (b), amino acid sequence with glycosylation patterns bolded and underlined (c), representative epitope consensus sequence (d), immunogenic regions with amino acid positions on S indicated from other informatic and seroreactivity studies and from IEBD data on B cell neutralising antibody response (see Table S5) (e), frequent mutations described in new variants of being monitored (VBM) and of concern (VOC) of SARS-CoV-2 S^122^, where the highly mutated Omicron (B.1.1.529) sublineages (designated with “O”) that have enhanced transmissibility and mutations that show differential (often escape from) neutralising antibody response (marked with “*”) are shown in (f) as *A1, A2, A3, A4, A5*—Omicron sublineages, BA1, BA2, BA3, BA4 and BA4 respectively.^2,71,99,109,122–126^.

*N* neutralising/protective antibodies shown, **, high IgG titre was associated with poor clinical outcome (development of pneumonia, needing care in the intensive unit or needing assisted pulmonary ventilation).

Epitopes S1.1 to S7 locating to the N-terminal region of S1, encompassed the antigenic sites against which antibody response was detected by others in COVID-19 patients^27,55–57,61,65^ and also its neutralising effects (Table 2 and Table S5). Interestingly, three epitopes (S1.1 to S1.3) were encompassing the anti-NTD supersite of S with neutralisation activity^66–68^. Four epitopes (S1.4 to S1.7) were identified in the receptor binding domain (RBD, amino acids 319 to 542) of SARS-CoV-2 S protein, spanning amino acid residues (319 to 542) that are involved in angiotensin-converting enzyme 2 (ACE2) binding^53^, and targeted by neutralising antibodies^69,70^ (Fig. 1, Fig. S2B). The epitope S1.5 overlapped at the I468 residue with the binding site of highly neutralising antibodies which showed good breadth against SARS-CoV-2 variants but not against BA.2.12.1 and BA.4/BA.5 Omicron sublineages with L452 mutations^71^. Additionally, epitopes S1.1, S1.3-S1.8, S1.10 and S2.2 encompassed antigenic regions with SARS-CoV-2 S neutralising activity (Tables 2 and S5) with S1.6 with overlapping in the E484 residue of the Omicron BA.1 escape mutant (E484A)^2^. A few of the resolved epitopes (S1.9, S1.10 and S2.5) included N-glycosylation sites (^72^ and Table 2). However, some data demonstrate that glycosylation is not essential for serorecognition of linear epitopes in spike upon COVID-19 infection^73^. Of note, some of the epitopes, including with neutralising/protective activity, embedded the common mutations of the emerging Omicron variants (Fig. 1, Tables 2 and S5).

Collectively, these data suggest that IgG antibody responses to distinct epitopes of SARS-CoV-2 S protein is common across the naïve population and reactivity to the same antigenic regions is detected by serostudies of COVID-19 patients (Table 2 and ref in the Table 2).

### Epitopes on SARS-CoV-2 S protein identified in COVID-19 naïve sera are linked to heterologous pathogens

**Figure 2:**
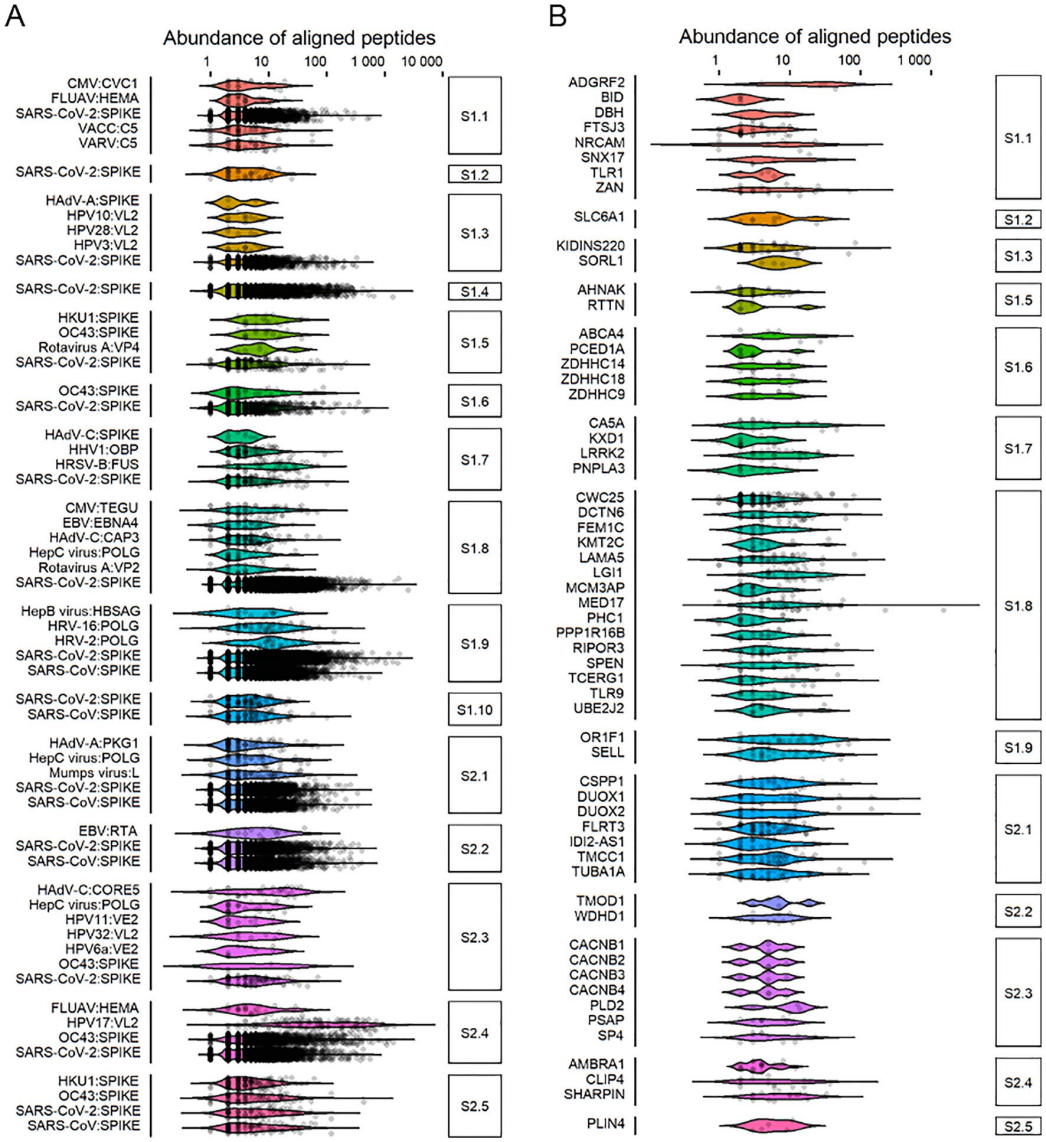
15 SARS-CoV-2 S protein epitopes mimic common viral protein antigens and self-proteins implicated in normal development and disease. Pathogen proteins and human proteome were accessed from UniProtKB and aligned with 13,500 most abundant IgG-bound 12-mer peptides containing one representative SARS-CoV-2 S protein epitope (from S1.1 to S2.5) using standalone BLAST with alignment criteria customised to short sequences. (**A,B)** Viral and human proteins mimicked by SARS-CoV-2 S protein epitopes are depicted on violin plots and were identified using *blastp* alignment analysis at E-value ≤ 0.05 (except for visualising SARS-CoV, SARS-CoV-2, HKU1, and OC43 alignments where E-value was not restricted). Each dot represents the relative abundance of IgG response to a peptide in one sample from the cohort of SARS-CoV-2 naïve subjects (n = 538). See Tables S3 and S4 for detailed information on pathogen and human protein alignments, full protein names and accession codes.

Next, we wanted to know whether cross-strain or cross-species immunity could be behind the observed epitope-specific pre-existing anti-SARS-CoV-2 S immunoreactivity. Sequence alignment analysis across human viral antigens resulted in frequent detection of other human coronaviruses (HCoVs, including SARS-CoV, OC43 and HKU) (Fig. 2A, Table S3). In addition, significant homology of the resolved S epitopes was also observed with common herpes-, papilloma-, and respiratory (including influenza) viral antigens (Table S3). For example, antigens of human cytomegalovirus (CMV) and of Epstein-Barr virus (EBV), shared significant similarity with peptides containing epitopes S1.8 and S2.2 (Fig. 2A, Table S3) and these epitopes similar to CMV and EBV seroprevalence were also associated with age (Fig. S4A). Diagnostic serology measurements confirmed CMV and EBV seropositivity in analysed samples (Fig. S5A). However, in samples with CMV and EBV serology findings differential epitope-specific anti-S antibody response was evident (Figs. S6, S7), suggesting that herpesviral antigens can be direct molecular mimics of S antigenic determinants or indirectly associated with the heterologous immunity towards SARS-CoV-2 S. Epitopes S1.10 and S2.5 showed higher antibody responses in CMV + samples of both Ctrl and Case groups when compared to CMV-samples (Fig. S6), while seroresponse to epitopes S1.6, S1.8, S1.9 and S2.1 was significantly higher (S1.8, S1.9, S2.5) or lower (S1.6) in EBV + samples of Case group when compared to EBV + Ctrl group (Fig. S7). High sequence similarity was also found between epitopes of SARS-CoV-2 S protein and antigens of influenza A H1N1 (FLUA), respiratory syncytial virus type B (HRSV-B), rhinoviruses 2/16 (HRV-2/16), adenovirus A type 12 (HAdV-A) and most frequent papillomaviruses (HPV6 and HPV11, Fig. 2A, Table S3). By using dot-ELISA, we independently validated the IgG antibody response detected by MVA at a peptide level to common epitopes of CMV glycoprotein B and EBV VCA p18 proteins^40^ (Fig. S5B). Collectively, our data conclude that heterologous immunity between epitopes of various common human viruses and SARS-CoV-2 S can be common.

Recent evidence also suggests that the existence of pre-COVID-19 autoimmunity plays a role in disease outcome^30,74^. Therefore, we focused on the human proteome and identified at least 63 human proteins with highly similar antigenic determinants to resolved epitopes of SARS-CoV-2 S (Fig. 2B, Table S4). Furthermore, analysis of the Human Protein Atlas expression data^42^ revealed that these proteins were differentially expressed with several of them displaying central nervous system and immune system specificity (Fig. S8). For example, epitope S1.3 mapped to kinase D-interacting substrate of 220 kDa (KIDINS220, Fig. 2B, Table S4), which has a crucial role in neuronal and cardiovascular development^75^. Relatedly, immune responses against epitope S1.3 were stronger in COVID-19 naïve men with heart disease (Fig. S4B). Epitope S1.7 showed mimicry to leucine-rich repeat serine/threonine protein kinase 2 (LRRK2; Fig. 2B, Table S4), which is associated with Parkinson’s and inflammatory bowel diseases^76,77^. High sequence similarity was found between epitope S2.2 and tropomodulin 1 (TMOD1) that is linked to synaptogenesis, chronic pulmonary disease and cancer^78^. Interestingly, epitope S2.3 that showed differential immunoreactivity in COVID-19 naïve smokers and women with hypertension (Fig. S4C,D) shared high sequence similarity with proteins associated with hypertension^79^, acute respiratory distress syndrome, periodontitis in smokers^80,81^, but also with proteins involved in the development of the nervous system^81^, and psychiatric disorders^82^ (Fig. 2B, Table S4). Collectively, our data show that distinct seroresponse to epitopes of SARS-CoV-2 S protein in COVID-19 naïve could accommodate cross-reactive targets of B cell response against both, viral pathogens and self-proteins.

### Epitopes on spike protein identified in COVID-19 naïve are differentially targeted by antibodies in COVID-19 diseased

**Figure 3:**
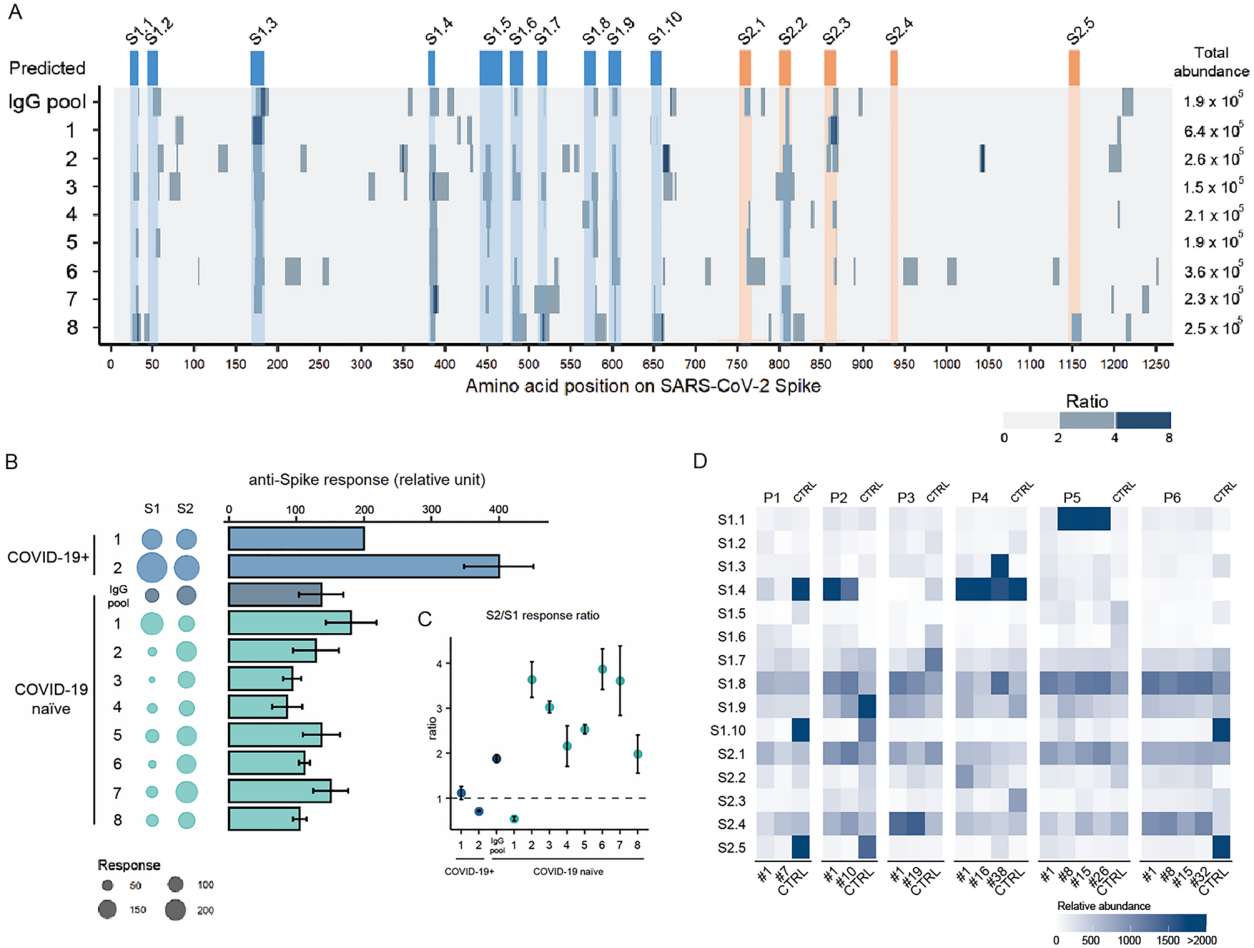
Differential antibody response to SARS-CoV-2 spike protein in COVID-19 diseased and naïve individuals. **(A)** Individual MVA immunoprofiles of antibody response to 15 epitopes of SARS-CoV-2 S in COVID-19 naïve samples shown as a ratio of specific vs random alignments. Ratios are visualised as centred moving averages across 9 amino acids. Y-axis—identifiers of samples, IgG pool refers to human pooled IgG sample, numbers 1–8 refer to COVID-19 naïve subjects (same as in **B,C**), Predicted—MVA-delineated epitopes on SARS-CoV-2 S; S1.1 to S1.10—epitopes on subunit 1 of S (dark blue); S2.1 to S2.5—epitopes on subunit 2 of S (orange colour); total abundance—calculated total abundance of IgG-bound peptides aligned to S per individual sample; colour bar (ratio)—ratio of alignment loads of specific vs random (scrambled) IgG-bound peptide sets. (**B)** Seroreactivity of COVID-19 naive (n = 8) and COVID-19 patients (n = 2) to spike protein subunits S1 and S2. 50 ng of SARS-CoV-2 S protein recombinant subunits S1 and S2 were immobilised on nitrocellulose slides and incubated with human serum/plasma samples to measure immunoreactivity to SARS-CoV-2 spike protein. Samples (n = 8) of selected subjects from COVID-19 naïve cohort (COVID-19 naïve 1–8) were used, alongside with the pooled human IgG sample (IgG pool, n = 2700 individual healthy donors, Sigma-Aldrich, # I4506). Samples (n = 2) taken at hospital intensive care admittance from patients (P1#1, P2#1) diagnosed with COVID-19 were included as positive controls for anti-spike immunoreactivity (COVID-19+*, *1–2). Dots represent dot-ELISA data normalised to the sample from patient 1 (P1#1) (COVID-19+) separately for S1 and S2 subunits (100 represents 100%). Bar plot shows the sum of average of dot-ELISA results for S1 and S2 from independent experiments (n = 3). Error bars represent summarised SEM from independent S1 and S2 results. (**C)** Scatter plot depicts average ratio of SARS-CoV-2 S2 and S1 signals in dot-ELISA experiments (n = 3) from (**B)**. Error bars represent SEM. (**D)** Heatmap shows the abundance of IgG-bound peptides containing the 15 epitopes of SARS-CoV-2 S protein in COVID-19 patients and controls. MVA immunoprofiles of serial samples from COVID-19 patients (n = 6) at different time points, where #1 is the sample taken at time of hospital admittance and “#n” where n designates the number of days from the first sample withdrawal. CTRL (n = 6) are age- and gender-matched healthy subjects selected from the cohort of 538 people. Relative abundance depicts the abundance of IgG-bound peptides containing the corresponding S epitopes, where values above 2000 are capped to 2000.

Next, we randomly picked samples (n = 8) from the COVID-19 naïve cohort with differential epitope profiles (Fig. 3A) along with the pooled IgGs of healthy subjects (n = 2700) (Fig. 3B,C, Table S1) to test for the presence of anti-S seroreactivity by dot-ELISA. Samples from patients from intensive care unit with severe COVID-19 disease (n = 2, COVID-19 +) were included for reference (Fig. 3B,C, Table S1).

The dot-ELISA signals detecting IgG response to recombinant spike subunits in both COVID-19 naïve and COVID-19 + samples were at similar value range (Fig. 3B, Fig. S9). Notably, anti-SARS-CoV-2 S protein seroreactivity was detected in all studied COVID-19 naïve samples (n = 9) (Fig. 3B) with more cross-reactivity to S2 subunit as compared to S1 (S2/S1 signal ratio > 1), although one naïve individual (#1) showed a reversed pattern (S2/S1 signal ratio < 1, Fig. 3C).

Next, we analysed samples from patients who had been admitted to the hospital with the COVID-19 diagnosis (Table S1). Detailed, epitope-specific analysis of time-series samples from SARS-CoV-2 infected patients (n = 6) and matched controls (n = 6) was performed using MVA. While reactivity to epitopes S1.1 and S1.3 increased during COVID-19 progression, reactivity to epitopes S1.8, S2.1 and S2.4 remained high across the studied time-window (Fig. 3D, Table S1). High reactivity to epitopes S1.1, S1.3, S1.4 (located in the RBD) and S1.8 was detected in three COVID-19 patients and in some COVID-19 naïve individuals, however the high antibody reactivity to S1.4 decreased in time of the COVID-19 patients’ stay in the hospital. Low immune reactivity to S1.10 and S2.5 was observed in COVID-19 diseased, wherein response to these epitopes in naïve was high (Fig. 3D). Overall, we show that epitopes on spike protein identified in COVID-19 naïve are differentially targeted by antibodies elicited in COVID-19 patients.

### Pathogenic epitopes on the S protein of SARS-CoV-2

**Figure 4:**
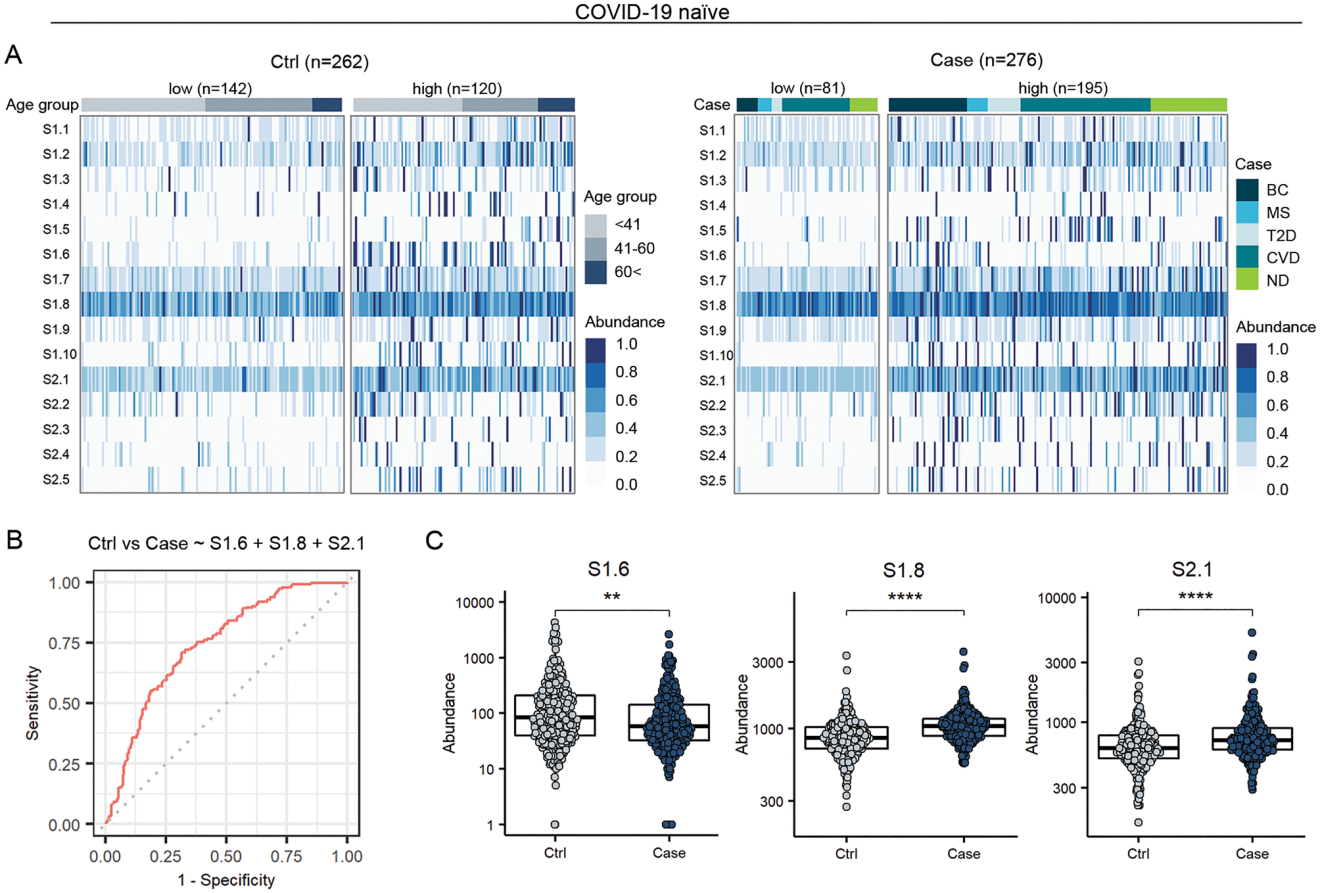
Antibody response to immunodominant epitopes on SARS-CoV-2 spike protein as predictors of ill health. **(A)** Individual immunoprofiles of SARS-CoV-2 S protein epitopes in COVID-19 naïve subjects, grouped into Ctrl (n = 262) or Case (with presented chronic diseases) (n = 276). “low” or “high”—subjects with relatively lower or higher overall response to S, calculated based on abundance of IgG-bound peptides containing the epitopes (S1.1 to S2.5) on SARS-CoV-2 spike protein (see “Methods”). x-axis—individual samples sub-grouped by Abundance (blue colour bar) where colour intensity shows individual abundance of IgG-bound peptides containing epitopes of spike normalised with 97.5th percentile value for visualisation. Age (grey colour bar) in Ctrl; y-axis—epitopes on Spike; *S1* epitopes on subunit 1 of S; *S2* on subunit 2 of S; COVID-19 naïve Case sub-groups: *BC* breast cancer (n = 57), *MS* multiple sclerosis (n = 20), *T2D* type 2 diabetes (n = 25), *CVD*—cardiovascular disease (n = 114), *ND*—neuropsychiatric disorders (n = 60). (**B)** Multivariable logistic regression analysis was used to describe the associations of epitope seroresponse predictors with the acute and/or chronic disease outcomes. Figure shows receiver operating characteristic (ROC) curve of using response to epitopes S1.6, S1.8, and S2.1 on training data (80% subset, i.e. 431/538 samples) for classifying Case (n = 276) vs Ctrl (n = 262). Area under curve (AUC) = 0.74 with 95% CI = (0.70…0.79). On validation with test set of 20% (107/538) samples, the select model classified samples into Case vs Ctrl with balanced accuracy of 62.0% for “low” group and 65.2% for “high” group (Fig. S10C). (**C)** Separately, antibody response to epitope S1.6 was identified as prevalent among Ctrl group subjects, whereas immune responses to epitopes S1.8 and S2.1 were prevalent among Case group in COVID-19 naïve cohort. Mann–Whitney U test, **p < 0.01; ****p < 0.0001. Group sizes: Ctrl (n = 262), Case (n = 276); abundance*—*abundance of IgG-bound peptides containing respective epitopes.

We investigated whether pre-existing antibody response to SARS-CoV-2 S epitopes in COVID-19 naïve people could be landmarks of ill-health. For that, COVID-19 naïve cohort was divided into two: a Case group (n = 276) of subjects with diagnosis of different acute and chronic conditions (cardiovascular disease (CVD), breast cancer (BC), multiple sclerosis (MS), type 2 diabetes (T2D), or neuropsychiatric disorders (ND)), and a Control group (n = 262) (Table 1). First, we observed that pre-existing high immune response to epitopes of the S protein was significantly prevalent in the Case group (χ^2^ test, ****p < 0.0001) (Fig. 4A and Fig. S10B), whereas the classification into low- or high- seroresponse groups was neither associated with age nor gender (χ^2^ test, ns p > 0.05, Fig. S10A). Correlation analysis showed no correlation between age of subjects and abundance of immune response as detected by MVA (Pearson R < 0.3, Table S6).

As Case group included a different proportion of “low” and “high” sub-groups compared to Control group (Fig. 4A), we developed multivariable logistic regression models by using 80% of the subset as a training data set to identify which pre-existing antibody epitopes on S were pathogenic and associated with serious health problems. We found that antibody response to epitopes S1.6, S1.8 and S2.1 was effective for identifying Case subjects with diagnosis of serious acute and chronic conditions (95% confidence interval (CI) for the area under the receiver operating characteristic (AUROC) was 0.697…0.790), sensitivity 71.0%, specificity 68.6% and the balanced accuracy 69.8%) (Fig. 4B). Separately, prior high antibody response to S1.6 was more frequent in Control group, whereas pre-existing response to S1.8 and S2.1 was more prevalent in Case group (Fig. 4C). Cross-validation of the model against independent testing dataset (20% subset of data) was equally accurate within both “low” and “high” groups, with balanced accuracy values of 62% and 65%, respectively (Fig. S10C). Further, we observed that pre-existing antibody response to some of these S-specific epitopes was associated with certain disease groups. For example, pre-existing response to epitope S1.8 was higher in subjects with hypertension (Fig. S4B). Overall, we suggest that prior seroresponse to a combination of three epitopes of S (S1.6, S1.8 and S2.1) may be used for predicting the underlying risk of aggravated immunopathology of acute/chronic condition due to or associated with exposure to SARS-CoV-2 S antigen.

## Discussion

### Seroreactive immunodominant epitopes on spike of SARS-CoV-2 in COVID-19 naïve

Here, we employed a high-resolution antibody response profiling technology (MVA) on a heterogenous cohort of COVID-19 naïve subjects including healthy subjects and people with one or more chronic or acute condition(s). Highly varied antibody response to SARS-CoV-2 and its emerging variants, in respect to antibody titres and neutralising activity, in the infected, vaccinated or people with hybrid immunity has been reported by several studies^83–86^. However, few of these studies have characterized the individual differences in the pre-existing antibody epitope repertoire. We identified 15 highly immunogenic epitopes characteristic to naïve populations in SARS-CoV-2 S protein localising in NTD, RBD, FP, HR1 and HR2 domains with notable heterogeneity among individuals (Figs. 1 and 4A). Our 3D analysis showed that majority of these were surface-exposed epitopes (Fig. S2) and encompassed antigenic regions determined by other studies underscoring the clinical relevance of the resolved 15 epitopes (Table 2). However, a notable difference between our findings and others (see Table 2 for references) was that we observed cross-reacting IgG reactivity in COVID-19 naïve individuals to epitopes (S1.4, S1.5, S1.6) in RBD that included residues of the binding sites of neutralising antibodies (Table S5). We observed that predominant seroreactivity in naïve sera was against antigenic determinants outside the RBD of SARS-CoV-2 S (Fig. 1), consistent with the increasing recognition of non-RBD neutralising antibody response^87^. Several of these epitopes concurred with the dominant linear epitopes that were identified by the study of mRNA vaccines as shared by vaccine naïve individuals and COVID-19 infected subjects^88^. Further, NTD-targeting antibodies with neutralising activities are of high interest (ref in^67^). Our epitope S1.3 locates to the N4 loop of the S protein, which is an antigenic site of monoclonal antibodies with potent neutralising activity directed to NTD^66^ thus suggesting that anti-S1.3 antibodies might interfere with anti-NTD supersite neutralising potential^89^. Several of the resolved epitopes within the S2 subunit overlapped with neutralisation determinants of SARS-CoV-2 S protein reported by others^21,43,45,52^ suggesting that there is quite substantial polyclonal neutralising potency towards SARS-CoV-2 in COVID-19 naïve sera. Of note, immunisation with S2 in mice has shown more potent neutralising antibody response than booster vaccines^90^. Given that recent molecular studies show the abundance of neutralising antibody targets on the SARS-CoV-2 S protein^91^, our data on pre-existing immunity highlights the need to correlate these findings on polyclonal neutralisation potency and its breadth with histories of infections and vaccinations^92–94^. Countries are likely to have distinct immune profiles because their histories of COVID-19 waves and vaccination rates differ, suggesting that these differences manifest in differential cross-recognition of SARS CoV-2 infections/vaccines at the level of binding and neutralising antibody-based immunity.

Many of the SARS-CoV-2 Omicron sublineages have evolved that carry distinct spike mutations and represent an antigenic shift resulting in escape from antibodies induced by previous infection or vaccination^95^. Our data show that some of these mutations (specifically, L452Q/R, E484K, F486V, N658S), frequently present in novel variants of concern^68,71^) are located within epitopes S1.5, S1.6, and S1.10 respectively (Fig. 1 and Tables 2 and S5). E484K has been shown to decrease the neutralisation ability of anti-spike antibodies tenfold^96–99^, L452Q/R and F486V are escape mutations in the RBD of Omicron sublineages from the cross-reactivity of neutralising antibodies, and N658S contributes to interference on hACE2 binding^71^. Cross-reactive antibodies targeting the dominant linear epitopes on SARS-CoV-2 S may contribute to neutralisation. Foremost, neutralising antibodies targeting the linear epitope 440–449^2^, such as REGN-10987 (Imdevimab)^100^, COV2-2130 (Cilgavimab, component of Evusheld)^101^ and LY-CoV1404 (Bebtelovimab^102^) were reported to neutralise BA.2 subvariants and BA.4/5, with LY-CoV14044 notably demonstrating high potency against all Omicron subvariants^71^. On the other hand, cross-reactive activity from the binding of antibodies to SARS-CoV-2 could contribute to the control of infection by antibody-dependent mechanisms^83^ and potentially amplify the damage that the virus causes to the body. The related mechanism involves antibody-dependent enhancement (ADE), a phenomenon in which non-neutralising/binding or sub-neutralising antibodies promote virus infection (rev in^103^). Most recently, early evidence of ADE in SARS-CoV-2 has begun emerging^104^. All these data further underscore the necessity for precise molecular characterisation of the effects of the pre-existing antibody on shaping the immunity to emerging SARS-CoV-2 infections and vaccines.

### Heterologous immunity landscape on SARS-CoV-2 S

Most recently, the question of whether immune history affects SARS-CoV-2 infection outcome has been replaced by the question to what extent pre-existing immunity plays a role. Similar to studies on influenza whereby the antibody response to older virus strains had profound and negative impacts on subsequent immunity^13^ cross-reactive antibody reactivity conferred by prior seasonal coronaviruses has widely been reported (ref in^20,105^). Higher titres of IgG against the HCoV-OC43 S protein were observed in patients with severe COVID-19^106^, concluding that such immunological imprinting by previous seasonal coronavirus infections negatively impacted on the antibody response against SARS-CoV-2 infection^107^. Similarly, the exposure to the antigenically shifted Omicron primarily leads to a recall of existing memory B cells specific for epitopes shared by multiple SARS-CoV-2 variants rather than by priming naïve B cells recognising Omicron-specific epitopes showing that previous SARS-CoV-2 infection history can imprint a profound negative impact on the subsequent protective immunity^86^.

In addition to cross-reactive epitopes with Omicron sublineages and also with endemic coronaviruses evidence of heterologous immunity between SARS-CoV-2 and pathogenic bacteria was reported^108^. Our study advances the concept showing that the cross-reactivity to epitopes of SARS-CoV-2 S protein with potential functional impacts could stem from the molecular mimicry with antigens of previously encountered other pathogens, including herpes-, papilloma-, adeno-, rhino-, influenza and other viruses (Fig. 2A). In good agreement with this, CMV seropositivity and age-related reduction in antibody titres against certain CMV antigens associated with the severity of SARS-CoV-2 infection^109^. Conversely, several other studies suggest cross-protection against COVID-19 incidence and severity from vaccines of influenza^28,110–112^ and of other pathogens (polio, HIB, MMR, Varicella, PCV13, and HepA–HepB)^28,110–113^. These mechanisms may include generation of cross-protective antibodies through molecular mimicry. Cross-reacting antibodies with SARS-CoV-2 proteins elicited by poliovirus^114^ and pneumococcal bacteria^115^ have been identified, whereas for the mumps virus (via the MMR vaccine), the cross-reactivity of the vaccine antigen (measles fusion glycoprotein) with RBD of SARS-CoV-2 spike was suggested^113^. Additionally, monoclonal antibodies against SARS-CoV-2 S RBD have been shown to cross-react with the Ebola glycoprotein and HIV-1 gp140^116^. Our data predict 15 cross-reactive SARS-CoV-2 spike-like epitopes in common pathogens (Fig. 2A). Although it is not clear how this pre-existing anti-pathogen humoral immunity impacts SARS-CoV-2 infections or vaccine efficacy, Heterologous Vaccine Intervention as a strategy against COVID-19 is advocated by governmental bodies as advantageous for populations with suboptimal response to vaccination (e.g., patients with altered immunocompetence)^117^. Altogether these findings along with ours illustrate how immunological imprinting from prior exposure, i.e., ‘original antigenic sin’, can strongly affect the response to novel antigens. Whether the 15 SARS-CoV-2 S epitopes presented here elicit cross-reactive antibody response with antigens of pathogens and/or of human origin needs further investigation by functional studies.

### Pathogenic epitopes on SARS-CoV-2 S

To our knowledge, this is a sole study to fine-map pre-existing antibody immunity to epitopes on SARS-CoV-2 S that associate with acute and/or chronic conditions like cardiovascular, neurological and oncological disease. Vastly different serological signatures to SARS-CoV-2 S that we detected in subjects with COVID-19 were also observed in healthy and people with chronic disease diagnosis suggesting a model where (recurrent) exposure to SARS-CoV-2 S-specific immune stimuli would progressively induce antibodies against certain epitopes landmarking chronic disease. We show the value of three epitopes on S as biomarkers to discriminate within COVID-19 naïve subjects between healthy and chronically diseased (with 95% CI, AUROC 0.69…0.79) (Fig. 4B and Fig. S10C). Ever since the first COVID-19 cases, immune-mediated manifestations have been reported^118^. A total of 55 long-term effects are associated with COVID-19^119^. Among these are myriad neurologic complications—including confusion, stroke, and neuromuscular disorders—which manifest during acute COVID-19 and related maladies lasting for months, and implicate immune dysfunction, including anti-neural autoimmune dysregulation^120^. Autoimmunity has become the hallmark of post-COVID syndrome and latent autoimmunity correlates with humoral response to SARS-CoV-2^74^. Here, we elaborate this aspect of findings further by our data showing that the resolved 15 epitopes on SARS-CoV-2 S protein share similarity with many human proteins of immune and nervous system origins (Fig. S8). Specifically, epitope S1.6 of RBD shares similarity with regions in highly expressed CNS proteins (Fig. 2B). Relatedly, H1N1 infection and the Pandemrix vaccine were found to be associated with narcolepsy^121^ by stimulating immune response targeting many cross-reactive autoantigens^41^. This suggests that conservation of antigenic sites across pathogen and human proteomes that we observed as epitopes on SARS-CoV-2 S protein in COVID-19 naïve individuals may have resulted (at least partially) from limited immune pressure for compatible immune fitness. However, further studies on epitopes of SARS-CoV-2 S are warranted, in particular in the settings of emerging strains and growing number of vaccines.

Collectively, our study provides evidence on the pre-existing immunity in COVID-19 naïve/unvaccinated individuals targeting 15 dominant epitopes on S protein. We show that this cross-reactive antibody response is boosted during SARS-CoV-2 infection in epitope-specific manner (Fig. 3D). Our findings are consistent with similar reports on pre-existing anti-SARS-CoV-2 S antibody immunity from others on cohorts with different genetic and demographic backgrounds^27,55–57,61,86^. We also show that pre-existing immunity on SARS-CoV-2 S shares epitope mimics associated with ill health. Overall, these data support the role of personal immune history with functional consequences to the diversity of antibodies elicited due to the phenomenon of heterologous immunity, i.e. back-boosting, i.e. immune imprinting, i.e. “original antigenic sin”^86,118^. The study lends further credence to MVA generated immunoprofiles as robust and generalisable.

### Study limitations

A weakness of the study is the scarcity of information on the study participants, with no available data on the immune history of their infections and vaccinations. Another caveat is the limited sample size of COVID-19 infected individuals which we used for validating the pre-existing seroreactivity patterns observed in naïve samples. Further studies will be necessary to determine what roles the resolved epitopes play in anti-SARS-CoV-2 immunity. Although the resolved epitopes were located on the exterior surface of the spike trimer by and spike-specific seroreactivity in naïve sera was confirmed independently by dot-ELISA using globular and denatured S subunits, it needs further analysis of the function of these antigenic sites of whether these target neutralising or binding antibodies. Although our data analysis results show differential anti-SARS-CoV-2 S epitope reactivity in herpesvirus positive/negative individuals, further studies are warranted to prove direct cross-reactivity between SARS-CoV-2 S and other pathogens. The unexpected finding of the SARS-CoV-2 S epitopes to embed potential pathogenic antigenic determinants will require further investigation in the future. Deciphering the biological role of the conservation of the heterologous immunity hot-spots on SARS-CoV-2 S may contribute to the design of future vaccines.

## Supplementary Information

Supplementary Information.

Supplementary Table S2.

Supplementary Table S3.

Supplementary Table S4.

Supplementary Table S5.

## Data Availability

The data are not publicly available due to containing information that could compromise research participant privacy/consent. Any materials that can be shared will be released via a material transfer agreement.

## Abbreviations

ACE2: Angiotensin-converting enzyme 2
ADE: Antibody-dependent enhancement
AUROC: Area under the receiver operating characteristic
BC: Breast cancer
CAD: Coronary artery disease
CMV: Human cytomegalovirus
CoV: Coronavirus
CVD: Cardiovascular disease
Dot-ELISA: Dot enzyme-linked immunosorbent assay
EBV: Epstein-Barr virus
FEP: First episode psychosis
HCoV: Human coronavirus
HLA: Human leukocyte antigen
HSV-1: Herpes simplex virus-1
HT: Hypertension
mAb: Monoclonal antibody
MI: Myocardial infarction
MS: Multiple sclerosis
MVA: Mimotope-variation analysis
ND: Neuropsychiatric disorder
NP: Neuropathic pain
RBD: Receptor binding domain
RU: Relative unit
S: SARS-CoV-2 spike glycoprotein
S1: S1 subunit of S
S2: S2 subunit of S
SZ: Schizophrenia
T2D: Type 2 diabetes

## Material availability

No new reagents or materials were generated as part of this study.
