# Supplementary material for: Differential patterns of cross-reactive antibody response against SARS-CoV-2 spike protein detected for chronically ill and healthy COVID-19 naïve individuals
Source: Sci Rep. 2022 Oct 7;12:16817. doi: 10.1038/s41598-022-20849-6 (PMC9540097; doi:10.1038/s41598-022-20849-6)
Supplement: Supplementary file 1 — Supplementary Information. [file 41598_2022_20849_MOESM1_ESM.docx]

**Supplementary Information**

**Table S1**. **Data of clinical cohorts analysed by MVA and dot-ELISA methods, related to Figure 3**. The serial samples from subjects (n=6, Patients 1-6) with COVID-19 diagnoses that were collected initially in the hospital emergency room (ER) and the consecutive samples in the stationary care unit during disease progression. Age-, gender- and ethnicity matched control samples (n=6) for COVID-19 cohort from independently chosen donors; IgG pool of 2700 individuals (Sigma-Aldrich, #I4506) and randomly selected samples (n=8) from the cohort of COVID-19 naïve individuals (n=538, Samples 1-8). ICD-10 codes are provided in round brackets. #+numbers represent days after ER sample withdrawal, where #1 represents the ER sample.

| Sample name in figures | Available data | Known diagnoses of sample donor | Type of sample | Sample procurement | Gender | Age |
| --- | --- | --- | --- | --- | --- | --- |
| Patient 1:  (2 timepoint samples - #1 and #7) | MVA;  Dot-ELISA | COVID-19 (U07.1), other viral pneumonia (J12.8), Type 2 diabetes (E11.8), hypertensive heart disease with heart failure (I11.0), mixed asthma (J45.8) | Plasma | March-May 2020 | F | 63 |
| Patient 1 CTRL | MVA | Healthy | Plasma | June 2010 | F | 63 |
| Patient 2:  (2 timepoint samples - #1 and #10) | MVA; Dot-ELISA | COVID-19 (U07.1), other viral pneumonia (J12.8), ARDS (J80), acute respiratory failure (J96.0) | Plasma | March-May 2020 | M | 57 |
| Patient 2 CTRL | MVA | Healthy | Serum | April 2011 | M | 56 |
| Patient 3:  (2 timepoint samples - #1 and #19) | MVA | COVID-19 (U07.1) | Serum | March-May 2020 | M | 42 |
| Patient 3 CTRL | MVA | Healthy | Plasma | June 2010 | M | 42 |
| Patient 4:  (3 timepoint samples - #1, #16 and #38) | MVA | COVID-19 (U07.1) | Serum | March-May 2020 | M | 63 |
| Patient 4 CTRL | MVA | Healthy | Plasma | June 2010 | M | 63 |
| Patient 5:  (4 timepoint samples - #1, #8, #15 and #26) | MVA | COVID-19 (U07.1) | Plasma | March-May 2020 | F | 58 |
| Patient 5 CTRL | MVA | Healthy | Serum | April 2011 | F | 58 |
| Patient 6:  (4 timepoint samples - #1, #8, #15 and #32) | MVA | COVID-19 (U07.1) | Plasma | March-May 2020 | M | 61 |
| Patient 6 CTRL | MVA | Healthy | Serum | January 2013 | M | 61 |
| IgG pool | MVA; Dot-ELISA | Negative for antibodies to HIV, HCV, HBsAg and syphilis | Purified IgG from pooled normal serum | January 2019 | NA | NA |
| Sample 1 | MVA; Dot-ELISA | Breast cancer (invasive ductal carcinoma) | Plasma | Sept 2010 | F | 64 |
| Sample 2 | MVA; Dot-ELISA | Myocardial infarction, HT | Serum | August 2015 | F | 72 |
| Sample 3 | MVA; Dot-ELISA | Myocardial infarction, diabetes, HT | Serum | April 2015 | M | 39 |
| Sample 4 | MVA; Dot-ELISA | CAD, ACS, HT, dyslipidemia,  diabetes, gingivitis | Serum | January 2016 | M | 59 |
| Sample 5 | MVA; Dot-ELISA | CAD ACS, HT, PD, alveolar bone loss, dyslipidemia | Serum | January 2016 | M | 63 |
| Sample 6 | MVA; Dot-ELISA | Healthy (no psychiatric, metabolic, cardiovascular or inflammatory disease) | Plasma | Dec 2018 | F | 19 |
| Sample 7 | MVA; Dot-ELISA | Healthy (no psychiatric, metabolic, cardiovascular or inflammatory disease) | Plasma | Dec 2018 | F | 40 |
| Sample 8 | MVA; Dot-ELISA | Healthy blood donor | Plasma | June 2010 | F | 26 |

*CTRL* – control; *IgG pool* – purified immunoglobulins from Caucasian American donors (Sigma-Aldrich # I4506); ARDS – acute respiratory distress syndrome; *HIV* – human immunodeficiency virus; *HCV* – hepatitis C; *HBsAg* – hepatitis B surface antigen; *CAD* – coronary artery disease; *ACS* – acute coronary syndrome; *HT* – hypertension; *PD* – periodontitis; *F* – female; *M* – male; *NA* – not available

**Table S2. Characteristics of 10 most abundant peptides sequenced in study cohort of 538 samples per each of the 15 defined spike epitopes, related to Methods.**

(as separate spreadsheet file)

**Table S3. Potential origin of SARS-CoV-2 spike protein cross-reactive antibodies in a naïve cohort, related to Figure 2.** 13 500 most abundant peptides corresponding to 15 spike epitopes were aligned to human-associated virus proteins (UniProtKB, accessed: 10.11.2020) that contained at least one predicted S epitope by using standalone BLAST+ blastp programme (see Methods). The table depicts alignments with the most relevant score with the criteria of E-value =< 0.05 or unlimited E-value in the case on SARS-CoV-2 and other coronaviruses.

(as separate spreadsheet file)

**Table S4. Potential cross-reactivity of SARS-CoV-2 spike protein reactive antibodies with human proteins, related to Figure 2.** 13 500 most abundant peptides corresponding to 15 spike epitopes were aligned to proteins from human reference proteome (UniProtKB, accessed: 10.11.2020) that contained at least one predicted S epitope by using standalone BLAST+ blastp programme (see Methods). The table depicts alignments with the best score with the criteria of E-value < 0.05. Orange indicates a corresponding HLA ligand epitope from www.iedb.org, whereas purple indicates a corresponding HLA ligand epitope from www.hla-ligand-atlas.org.

(as separate spreadsheet file)

**Table S5. SARS-CoV-2 Spike neutralising epitopes as reported in Immune Epitope Database that overlap with epitopes, related to Table 2.** SARS-CoV-2 spike neutralising epitopes were accessed on 11.07.2022 with the following criteria: Organism – SARS-CoV2 (ID:2697049); Antigen – Spike glycoprotein (P0DTC2); B cell Assay – Outcome “Positive” and “Neutralization”.

(as separate spreadsheet file)

**Table S6.** **Pearson correlation between age and abundance of immune response as detected by MVA.** The following information is given in columns: unique identification (a), amino acid position (b), amino acid sequence with glycosylation patterns bolded and underlined (c), representative epitope consensus sequence (d), Pearson correlation index (e).

| Epitope identification (a) | Amino acid position (b) | Sequence (c) | Representative epitope (d) | Correlation with age (e) |
| --- | --- | --- | --- | --- |
|  |  |  |  |  |
| S1.1 | 26-34 | PAYTNSFTR | NSF.R | R = 0.056  p=0.2 |
| S1.2 | 47-58 | VLHSTQDLFLPF | V..S..D…P | R = 0.059  p=0.17 |
| S1.3 | 170-185 | YVSQPFLMDLEGKQGN | L..K.GN | R = 0.014  p=0.74 |
| S1.4 | 384-390 | PTKLNDL | PTKL..L | R = 0.037  p=0.4 |
| S1.5 | 445-471 | VGGNYNYLYRLFRKSNL-KPFERDISTE | K….DI.T | R = 0.18  p=0.0000033 |
| S1.6 | 481-495 | NGVEGFNCYFPLQSY | N.VE.F | R = -0.18  p=0.0000037 |
| S1.7 | 514-523 | SFELLHAPAT | S…LH…T | R = 0.079  p=0.071 |
| S1.8 | 570-582 | ADTTDAVRDPQTL | PQTL | R = 0.15  p=0.00077 |
| S1.9 | 599-612 | TPGT**N**TSNQVAVLY | GTN.S | R = -0.056  p=0.2 |
| S1.10 | 650-660 | LIGAEHV**N**NSY | L..A…..SY | R = 0.035  p=0.42 |
| S2.1 | 757-768 | GSFCTQLNRALT | T.LNR | R = -0.052  p=0.23 |
| S2.2 | 804-815 | QILPDPSKPSKR | I.P…KP | R = -0.037  p=0.39 |
| S2.3 | 858-869 | LTVLPPLLTDEM | V.P.L…E | R = -0.0095  p=0.83 |
| S2.4 | 937-944 | SLSSTASA | SL.S…A | R = -0.0095  p=0.83 |
| S2.5 | 1151-1161 | ELDKYFK**N**HTS | EL….K...S | R = 0.035  p=0.42 |


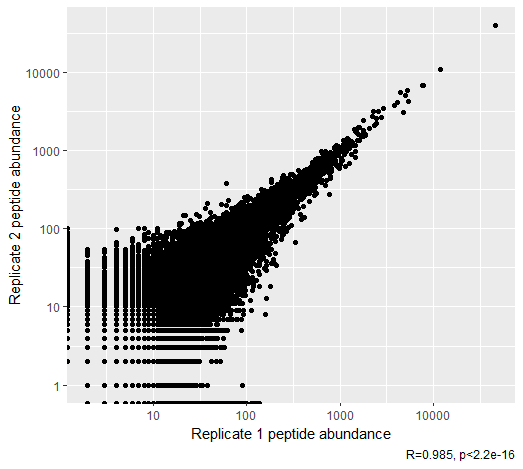


**Figure S1. A typical Pearson correlation (*R=0.985*) of peptide antigen profiles between two independent MVA studies confirms reproducibility of MVA analyses, related to Methods.** R programming language and package “ggpubr” was used to calculate correlation and perform hypothesis test on the correlation coefficient (p < 2.2e-16), and package “ggplot2” was used to visualise the results^1,2^.


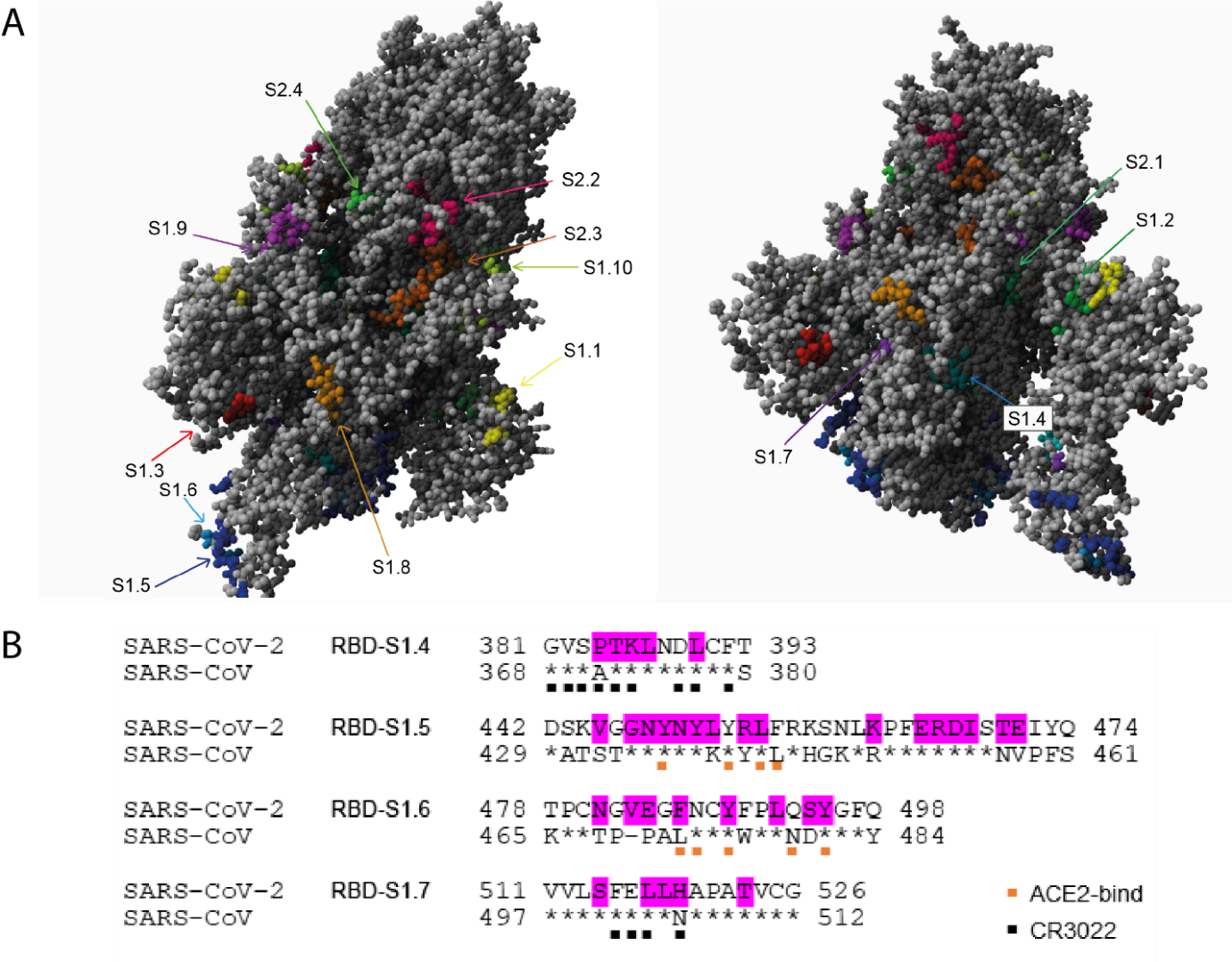


**Figure S2. 3D structure analysis of SARS-CoV-2 spike protein trimer revealed that majority of the MVA-delineated epitopes were on the surface and epitopes located in the RBD were conserved in SARS-CoV-2 and SARS-CoV S proteins, related to Figure 1 and Table 2. A|** SARS-CoV-2 S ectodomain trimer structures (6VYB) were taken from <https://www.rcsb.org/structure/6VYB> (date accessed 20.05.20) and analysed using molecular visualisation software YASARA View (http://www.yasara.org). Colour-codes indicate localisation of epitopes on the surface of the ectodomain SARS-CoV-2 S protein trimer. Epitope S2.5 could not be determined as the C-terminal sequence of SARS-CoV-2 S protein was not part of the 6VYB model. **B|** The degree of conservation for some resolved epitopes in SARS-CoV and SARS-CoV-2 S proteins is relatively high. Identity for epitope S1.5 that covers 27 amino acids is ~ 59.3%; identity for epitope S1.6 that encompasses ACE2 binding half-site is 60%. *Magenta* - exact matching amino acids in RBD of SARS-CoV-2 S to MVA epitopes. *Asterisks (*)* - amino acids on SARS-CoV-2 S that are identical with SARS-CoV S. *Hyphen (-)* – absence of amino acid. *Orange squares* - amino acids important for binding of SARS-CoV-2 S to human ACE2 protein^3,4^, *black squares* - amino acids important for binding of anti-spike neutralising CR3022 antibodies^3,5^.


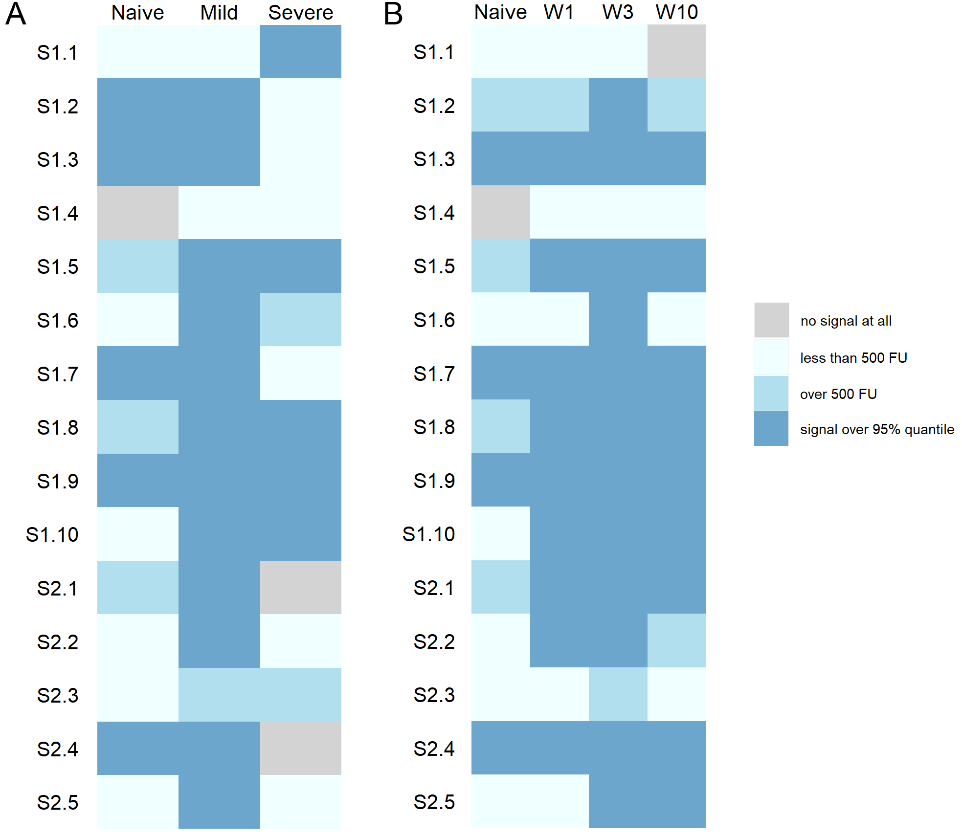


**Figure S3**. **The seroreactivity against 15 SARS-CoV-2 spike (S) epitopes of naïve and COVID-19 patients’ samples as measured in SARS-CoV-2 spike whole proteome microarray reported by Schwarz et al., 2021, related to Figure 1 and Table 2**. Raw data of PEPperCHIP® SARS-CoV-2 Proteome Microarray (PEPperPRINT GmbH, Germany), comprising the whole proteome of SARS-CoV-2 Wuhan-hu-1 isolate, IgG seroreactivity assay as reported by Schwarz et al., 2021^6^ is depicted. The assay included 7 healthy COVID-19 negative samples (*Naïve*), 9 samples of mild COVID-19 case (*Mild*) and 7 samples of severe COVID-19 case (*Severe*) (panel A). The 9 patients with mild COVID-19 were also included in a longitudinal study where blood was collected at disease progression of 1 week (*W1*), three weeks (*W3*) and 10 weeks (*W10*) (panel B). Fluorescence signals from peptides in SARS-CoV-2 IgG reactivity assay that encompassed the 15 spike epitopes (*y-axis*) delineated in the current study from 6 groups (*on-top of panel*, either *Naïve*, *Mild*, *Severe*, *W1*, *W3* or *W10*) were included in the analysis. The fluorescence intensity signal units of spike peptides are coded as “less than 500 FU” when at least one sample in the allotted group had a fluorescence signal over 0 but under 500 FU, “over 500 FU” when at least one sample in the allotted group had a fluorescence signal over 500 FU, but under 95% quantile and “signal over 95% quantile”, when at least one sample in the allotted group had a fluorescence signal over the threshold of 95% quantile (e.g. over 1044.75 FU for peptide signals in panel A and over 1102.875 FU for peptide signals in panel B). “no signal at all” states that no sample in the allotted group had a fluorescence signal over 0.


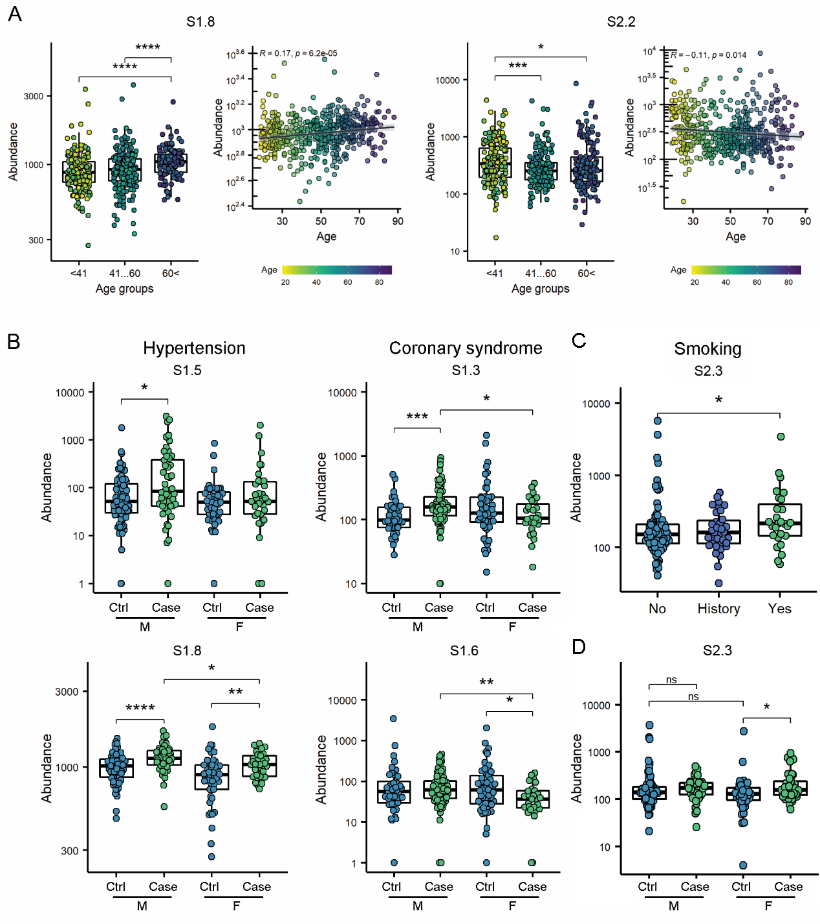


**Figure S4. Antibody response to specific epitopes on SARS-CoV-2 spike protein associate with age and comorbid conditions of hypertension, coronary syndrome, and cigarette smoking, related to Figure 2, 4. A|** Abundance and correlation plots of epitope-containing peptides across different age groups**.** *Age groups*:<41 (n=186), 41…60 (n=188), 60< (n=150). Pearson correlation coefficients (*R*) with p-values and *black line* showing trends across age groups with confidence limits (95% CI, *grey highlight*). Calculated non-adjusted p-values: S1.8 -1.9x10^-8 *(<41 vs 60<*) and 3.7x10^-5 (*41…60 vs 60<*), S2.2 - 0.023 (*<41 vs 60<*) and 0.00038 (*<41 vs 41…60*) (Mann-Whitney U test, p-values not adjusted for multiple comparisons). **B|** Epitope associations with hypertension and coronary syndrome. On boxplots, *y-axis* - abundance of IgG-bound peptides containing the epitope. *M* – male, *F –* female. Group sizes: *Hypertension*: Ctrl – M (n=71), F (n=43); Case – M (n=48), F (n=33); *Coronary syndrome*: Ctrl – M (n=42), F (n=51); Case – M (n=79), F (n=27). Mann-Whitney U test, ns p>0.05, * p<0.05, ** p<0.01, *** p<0.001, **** p<0.0001, p-values not adjusted for multiple comparisons. Calculated non-adjusted p-values: *Hypertension:* S1.5 - 0.012 (*Ctrl M vs Case M*), S1.8 - 9.7x10^-5 (*Ctrl M vs Case M*), 0.0066 (*Ctrl F vs Case F*), and 0.013 (*Case M vs Case F*). *Coronary syndrome:* S1.3 - 0.00037 (*Ctrl M vs Case M*) and 0.014 (*Case M vs Case F*), S1.6 - 0.0035 (*Case M vs Case F*) and 0.045 (*Ctrl F vs Case F*). **C|** Epitope S2.3 association with smoking. On boxplots, *y-axis* - abundance of IgG-bound peptides containing the epitope. Group sizes: “*No*” - non-smokers (n=94); “*History*” - previous history of smoking; (n=35); “*Yes*” - active cigarette smokers (“Yes”) (n=27). Mann-Whitney U test, * p<0.05. **D|** Epitope S2.3 associations with hypertension and gender in MI and CAD sub-cohorts. On boxplots, *y-axis* - abundance of IgG-bound peptides containing the epitope. Mann-Whitney U test, ns p>0.05, * p<0.05, p-values not adjusted for multiple comparisons. *Ctrl* – no hypertension, *Case* – hypertension, *M* – male; *F* – female. Group sizes: Ctrl (n=122), Case (n=81). Ctrl - M (n=71), F (n=43); Case - M (n=48), F (n=33).


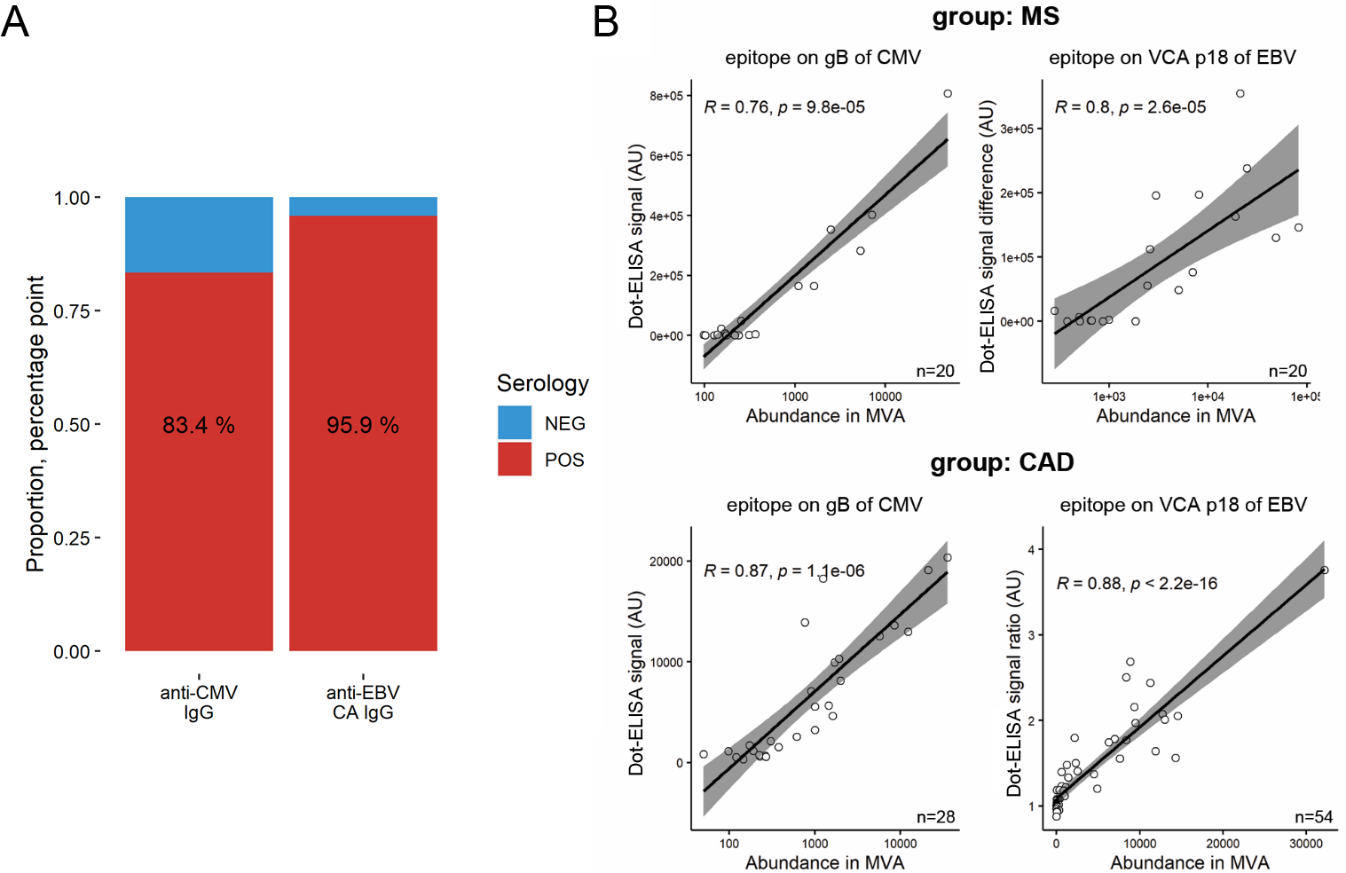


**Figure S5. Serology results of antibody response to common human herpesviruses cytomegalovirus (CMV) and Epstein-Barr virus (EBV) within the clinical cohort, related to Figure 2. A|** Altogether serum or plasma samples of 199 subjects from the clinical cohorts of healthy donors (n=83), multiple sclerosis (MS, n=20), or coronary artery disease (CAD, n=96) were analysed for anti-CMV IgG antibodies with commercially available ELISA method (ISO/IEC 17025:2017 accredited). 83.4% of the tested subjects were positive for anti-CMV IgG antibodies. Similarly, a set of serum or plasma samples of 241 subjects from the clinical cohorts of healthy donors (n=19), MS (n=20), first episode psychosis (FEP, n=60), schizophrenia (SZ, n=46), or CAD (n=96) were analysed for anti-EBV-CA IgG antibodies with a commercially available ELISA method (ISO/IEC 17025:2017 accredited). 95.9% of the tested subjects were positive for anti-EBV-CA IgG antibodies. *Y-axis* – proportion (as percentage points) of subjects, *Serology* – ELISA method identified subjects as either negative (NEG) or positive (POS) for anti-viral antibodies, *anti-CMV IgG*– tested for anti-CMV IgG antibodies, *anti-EBV CA IgG* – tested for anti-EBV-CA IgG antibodies. **B|** Mimotope variation analysis (MVA) results of immunoreactivity to specific epitopes were validated in independent dot-ELISA experiments in the clinical cohorts of MS or CAD. High response to epitope on gB protein of CMV (glycoprotein B; 70ETI**Y**NT**TL**K**Y**80) or VCA p18 protein of EBV (the viral capsid antigen p18; 161GGQ**P**HD**T**A**PR**GARKK175) observed by MVA was strongly correlated with high seroreactivity measured in dot-ELISA experiments. Spearman correlation analysis of dot-ELISA values of gB CMV or VCA p18 of EBV in the tested samples with abundance of similar IgG-bound peptide epitopes on MVA immunoprofiles. *y-axes* – dot-ELISA values in arbitrary units (AU) (dot-ELISA signal – as measured against slide background, dot-ELISA signal difference – calculated as difference of signals between specific and control epitope, dot-ELISA signal ratio – calculated as ratio of signals between specific and control epitope); dot-ELISA signal – signal as measured against background intensity *x-axes* – peptide epitope abundance in MVA immunoprofiles; *R* – Spearman correlation coefficient; *p* - correlation p-value.


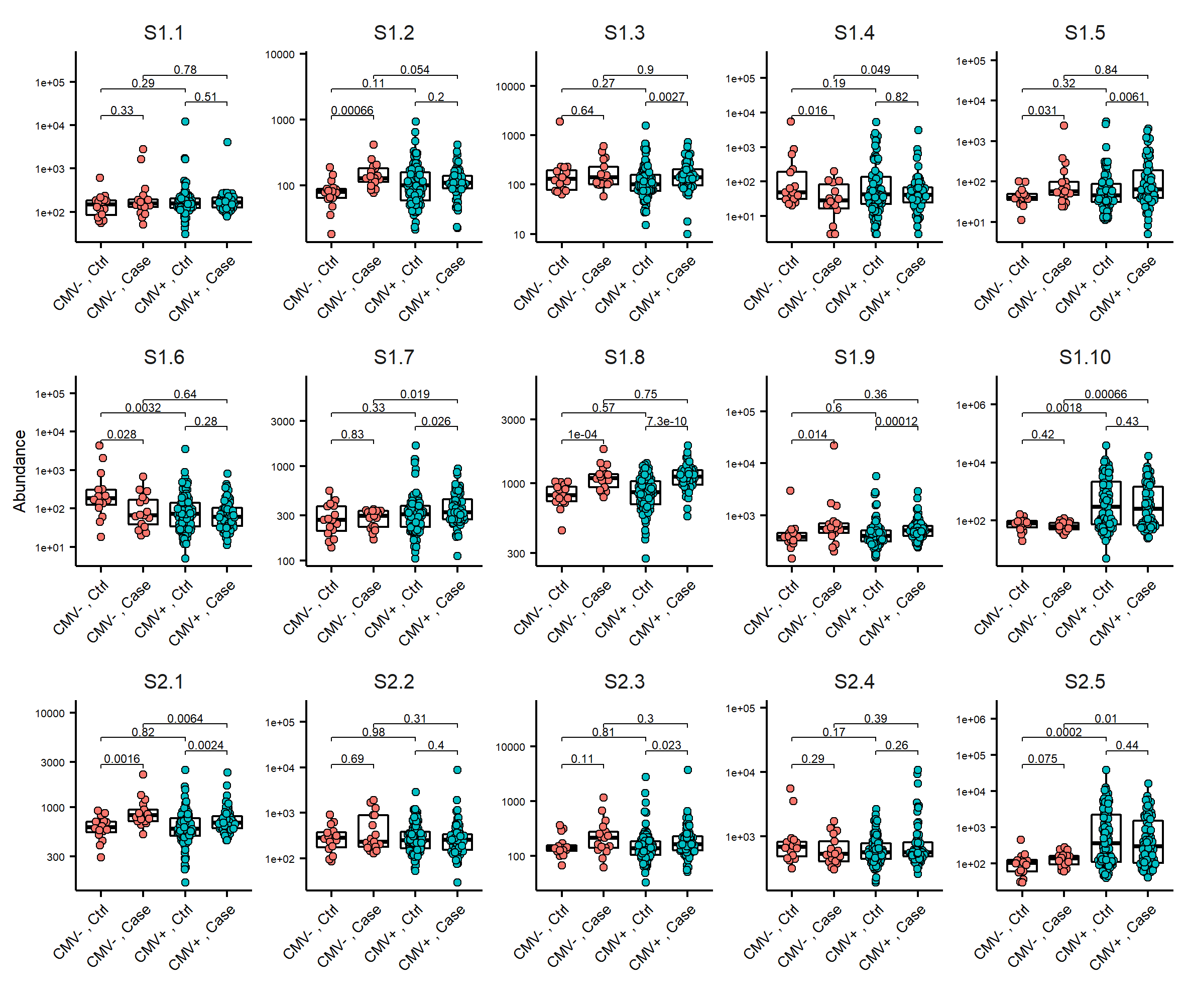


**Figure S6.** **Anti-CMV serology status, either negative (-) or positive (+) for IgG antibodies, associated with immune response to epitopes on spike (S) within COVID-19 naïve cohort**, **related to Figure 2**. Mann-Whitney U test, p-values shown on figures are not adjusted for multiple comparisons, *y-axes* – abundance of IgG-bound peptides containing given epitopes on S. *CMV-* - negative CMV serology, *CMV+* - positive CMV serology, *Ctrl* (n=115) – healthy blood donors (n=83) and control samples of CAD sub-cohort (n=32); *Case* (n= 84) – case samples of MS (n=20) and CAD (n=64) sub-cohorts. Group sizes: *CMV-, Ctrl* (n=16); *CMV-, Case* (n=17); *CMV+, Ctrl* (n=99), *CMV+, Case* (n=67).


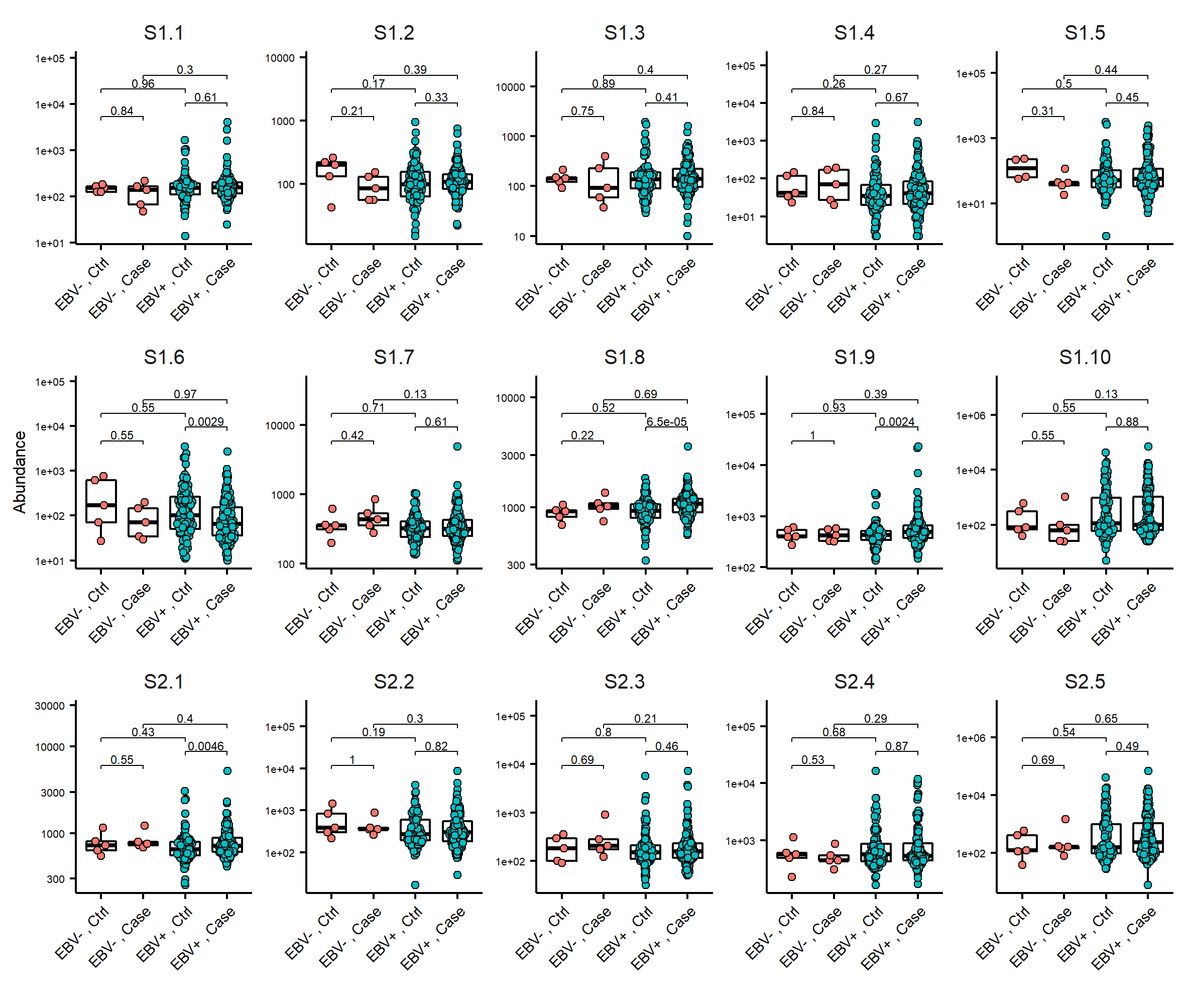


**Figure S7.** **Anti-EBV serology status, either negative (-) or positive (+) for IgG antibodies, associated with immune response to epitopes on spike (S) within COVID-19 naïve cohort, related to Figure 2.** Mann-Whitney U test, p-values show on figures are not adjusted for multiple comparisons, *y-axes* – abundance of IgG-bound peptides containing given epitopes on S. *EBV-* - negative EBV serology, *EBV+* - positive EBV serology. *Ctrl* (n= 97) – healthy blood donors (n=19), and control samples of CAD (n=32), SZ (n=16) and FEP (n=30) sub-cohorts; *Case* (n= 144) – case samples of MS (n=20), CAD (n=64), SZ (n=30) and FEP (n=30) sub-cohorts. Group sizes: *EBV-, Ctrl* (n=5); *EBV-, Case* (n=5); *EBV+, Ctrl* (n=92), *EBV+, Case* (n=139).


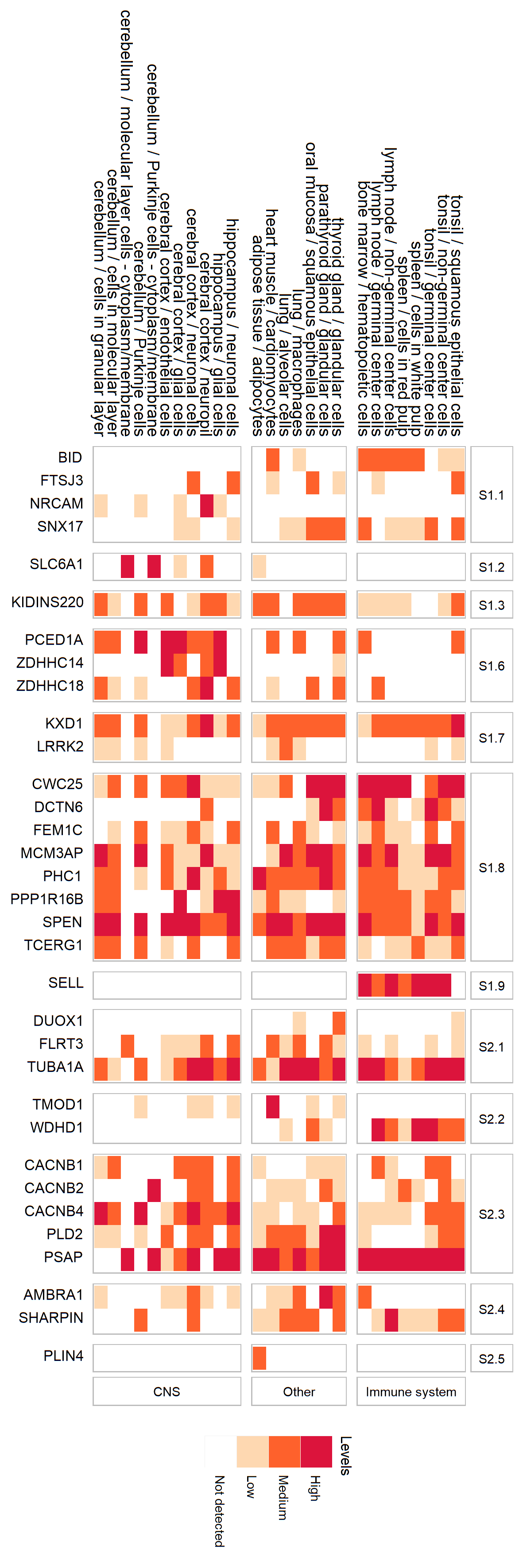
**Figure S8**. **Peptides mimicking 15 epitopes of SARS-CoV-2 spike protein align with human proteins associated with normal development or implicated in disease, related to Figure 2.** Heatmap visualisation of protein expression levels in normal tissues and cells of human proteins (data from Human Protein Atlas v. 20.0^7^), that aligned with SARS-CoV-2 S protein epitope containing peptides (E-value ≤ 0.05). White areas in heatmap refer to undetected expression levels of proteins in corresponding tissue or cell**.** Left-most (*y-axis*) annotations describe tissue and cell-type origin of expression data. Bottom *x-axis* denotes the gene names of proteins that aligned with peptides as referenced in UniProtKB database (**Table S4**), while top-most *x-axis* denotes the corresponding S protein epitopes.


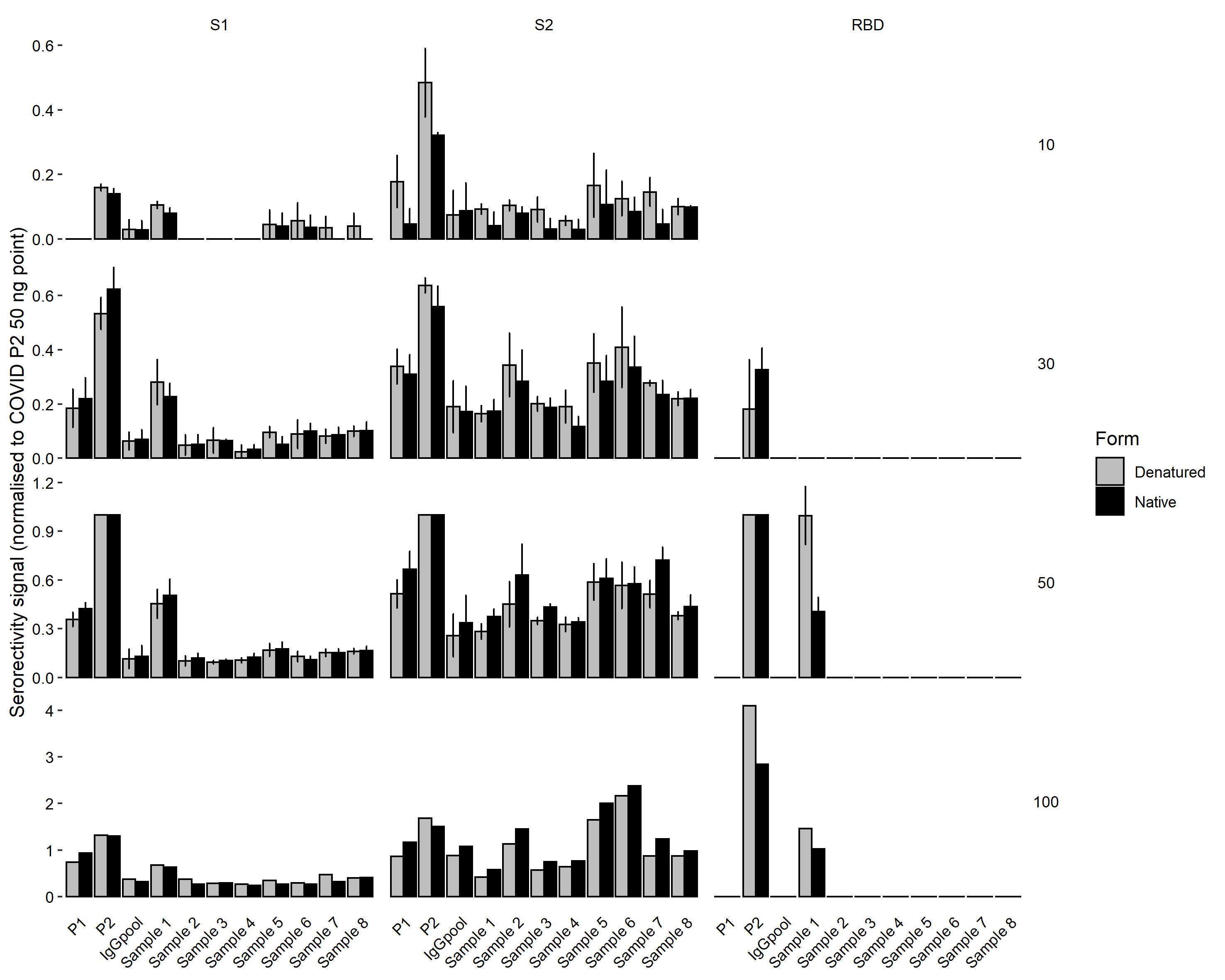


**Figure S9. Immune response against SARS-CoV-2 spike protein subunits in COVID-19 diseased and naive cohorts, related to Figure 3.** Native and denatured forms (*Form*) of recombinant spike (S) protein subunits S1, S2 and RBD were blotted onto nitrocellulose membranes (in amounts shown on right-side *y-axis* (either *10*, *30*, *50* or *100* ng) and incubated with 1:50 dilutions of two COVID-19 patient samples (ICD-10 code U07.1, *P1-P2*), IgG pool (pooled normal sera of ~2700 healthy donors (Sigma-Aldrich, #I4506, 10 mg/ml)) and selected SARS-CoV-2 sera/plasma samples (n=8) from the naïve cohort (n=538) for 4h at room temperature. Recombinant proteins were incubated with 4M urea at room temperature for 1h to denature the globular form of the proteins. Immunoreactions were visualised with CSA II System amplification kit (Agilent). Data is represented as a proportion of seroreactivity values normalised to the 50 ng dot signals of the respective subunit forms of S protein in a COVID-19-positive sample (P2). Error bars represent SEM from three independent dot-ELISA analyses (n=3), with the exception of RBD (*30* ng) and S1-S2 (*10* ng) experiments where SEMs of 2 experiments are shown. For *100* ng spike subunit dot-ELISA experiments n=1.


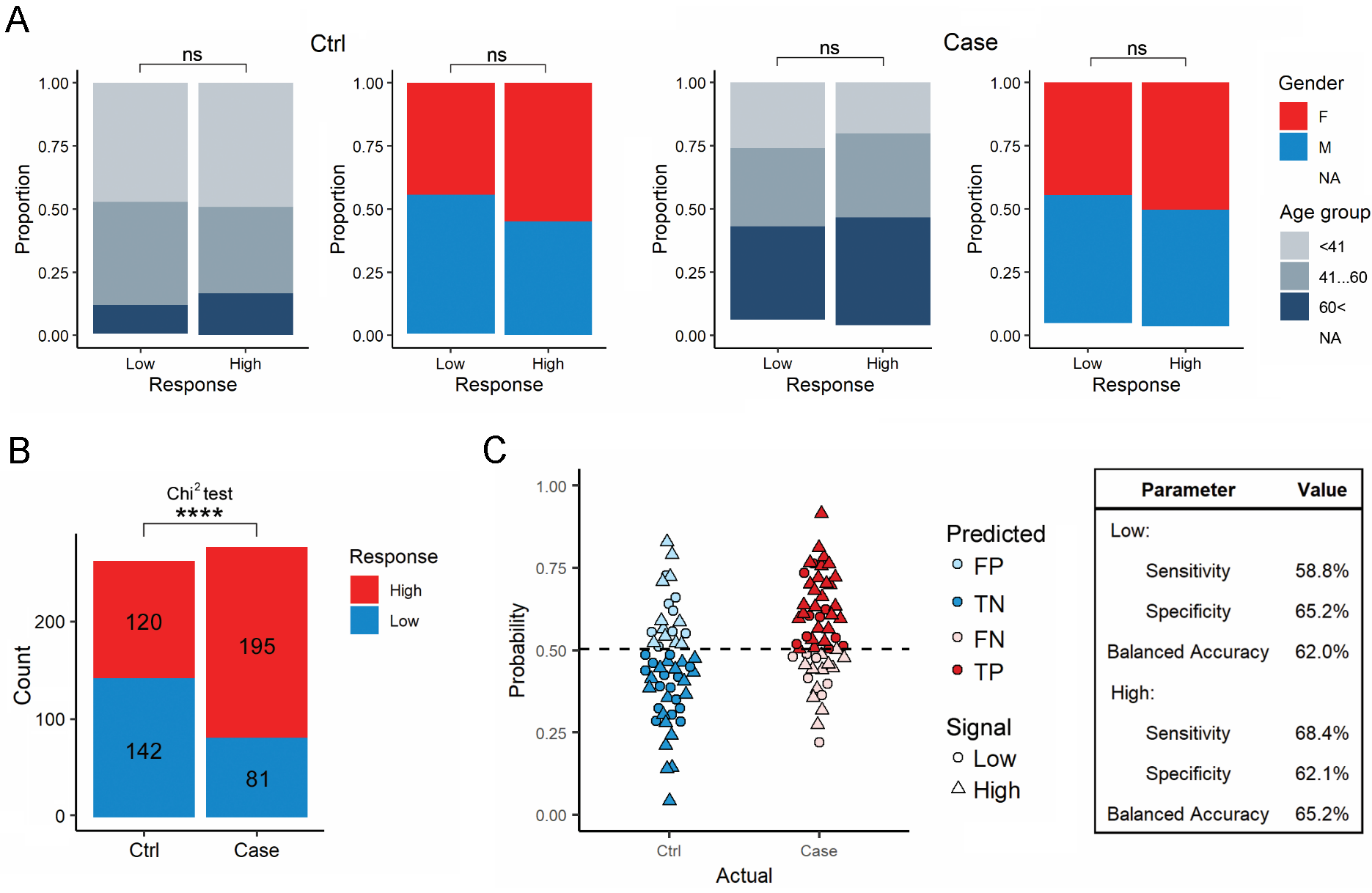


**Figure S10. Antibody response to embedded immunodominant epitopes on SARS-CoV-2 spike protein as predictors of ill health, related to Figure 4. A|** *Gender* and *Age* were not significantly associated with “*Low*”- or “*High*”- immunoreactivity to SARS-CoV-2 S protein in control (*Ctrl*) and *Case* groups. Chi^2^ test, *ns* - p-value > 0.05. *Proportion* – proportion of subjects from an age or gender group within the whole group. *M* – male, *F* – female. **B|** The proportion of subjects with a relatively higher immune response (*High*) to SARS-CoV-2 S epitopes was significantly bigger in the *Case* group of subjects with various chronic diseases as compared with the *Ctrl* group. Sub-group sizes are shown on the figure. Chi^2^ test, **** p<0.0001. **C|** Multiple logistic regression model of three epitopes of SARS-CoV-2 S protein tested on independent validation set classifies the clinically heterogenous *Case* group from *Ctrl* in COVID-19 naïve subjects. **A-C|** Abundance values of 15 epitopes were calculated for each sample in the COVID-19 naïve cohort (n=538) and normalised per epitope with the 97.5% percentile values (to allow for comparison of epitopes) (resulting in value range 0…1). Subjects with a relatively higher response (normalised value > 0.5) to at least two epitopes on S protein (of the 15) were grouped into the “high” group, whereas others to the “low” group. Classification results (*left*) of model on validation data (20% subset) with numerical accuracy parameters (*right*). Accuracy parameters were calculated separately for “low”- and “high”- response groups (here *Signal)*. *Probability* – calculated probability of a subject for being classified into “Case” group by multiple logistic regression model (see **Figure 4**). *Actual* – classification of the subject in the validation set. *Predicted*: *FP* – false positive, *TN* – true negative, *FN* – false negative, *TP* – true positive.

**Supplement References**

1. Kassambra A. ggpubr: ‘ggplot2’ Based Publication Ready Plots. . R package version 040. 2020.

2. Wickham, H. *ggplot2: Elegant Graphics for Data Analysis*. (Springer-Verlag, 2009). doi:10.1007/978-0-387-98141-3.

3. Yuan, M. *et al.* A highly conserved cryptic epitope in the receptor binding domains of SARS-CoV-2 and SARS-CoV. *Science* **368**, 630–633 (2020).

4. Chowdhury, R. & Maranas, C. D. From directed evolution to computational enzyme engineering—A review. *AIChE Journal* **66**, e16847 (2020).

5. ter Meulen, J. *et al.* Human Monoclonal Antibody Combination against SARS Coronavirus: Synergy and Coverage of Escape Mutants. *PLoS Med* **3**, (2006).

6. Schwarz, T. *et al.* SARS-CoV-2 Proteome-Wide Analysis Revealed Significant Epitope Signatures in COVID-19 Patients. *Frontiers in Immunology* **12**, 765 (2021).

7. Uhlén, M. *et al.* Tissue-based map of the human proteome. *Science* **347**, 1260419 (2015).
